# Artificial Intelligence in Renal Imaging: A Multi-Dataset Study for Kidney Disease Classification

**DOI:** 10.3390/biomedicines14051105

**Published:** 2026-05-14

**Authors:** Berçem Afşar Karatepe, Burak Tasci

**Affiliations:** 1 Internal Medicine Department, Elazığ Fethi Sekin City Hospital, 23280 Elazig, Türkiye; 2 Vocational School of Technical Sciences, Firat University, 23119 Elazig, Türkiye; btasci@firat.edu.tr

**Keywords:** deep learning, kidney disease classification, cross-institutional validation, attention mechanisms, medical imaging, dual-path neural networks

## Abstract

**Objectives:** To develop and rigorously evaluate a Hybrid Multi-Path Attention Convolutional Neural Network (HMPA-CNN) for the classification of kidney diseases across heterogeneous institutional datasets and imaging modalities. **Materials and Methods:** The proposed HMPA-CNN employs dual parallel pathways to disentangle spatial (3 × 3 convolutions) and textural (5 × 5 convolutions) representations, followed by attention-based feature recalibration and gated fusion. Performance was assessed on five geographically distinct datasets comprising 29,148 CT and MRI images collected from Turkey, Bangladesh, Jordan, Iraq, and publicly available international sources. The evaluation framework included three-class tumor discrimination, four-class renal pathology classification, six-class tumor subtyping, binary kidney stone detection, and chronic kidney disease (CKD) assessment under cross-modality conditions. **Results:** The model achieved 99.76% overall accuracy on the KidneyNeXt three-class dataset, 99.96% on the four-class multi-institutional CT dataset, and 99.74% on the independent Jordan cohort under a four-class configuration. In the more granular six-class tumor subtyping task, overall accuracy was 96.36%. The same architecture achieved 93.85% overall accuracy on the MRI-based CKD classification task, suggesting that the framework can be adapted to a different imaging modality. Across most classification tasks, specificity exceeded 99%, with benign–malignant misclassification remaining below 2%. Performance declined to 91.96% for kidney stone detection, reflecting the intrinsic difficulty of small-object localization in axial CT images. **Conclusions:** The dual-path architecture consistently preserved high discriminative performance across institutions, diagnostic granularities, and imaging modalities. Its stable specificity and low benign–malignant confusion suggest potential utility as a supportive tool within renal imaging workflows, particularly for screening and structured diagnostic assistance. Clinically, benign–malignant misclassification is the most critical error, as it may delay oncologic evaluation or lead to unnecessary follow-up. Therefore, the model should be used as a decision-support tool rather than an autonomous diagnostic system. Further prospective validation is required to determine its impact in routine clinical practice.

## 1. Introduction

Kidney diseases comprise a clinically diverse spectrum of conditions that collectively impose a substantial global health burden [1]. Chronic kidney disease, renal calculi, cystic disorders, and renal tumors frequently present with non-specific or overlapping symptoms, particularly in early stages, delaying diagnosis and narrowing therapeutic windows [2,3]. In this setting, timely and accurate identification of renal pathology directly influences treatment selection, surveillance strategies, and long-term functional outcomes.

Among renal pathologies, renal tumors present a distinct diagnostic challenge [4]. A major clinical challenge is distinguishing benign from malignant renal masses, as this distinction directly influences treatment selection, preservation of renal function, and patient prognosis [5,6]. The absence of symptoms in renal stones and cysts does not prevent their progression toward obstruction or infection or permanent damage to the kidney tissue when left untreated. Clinical practice, therefore, requires diagnostic systems that can detect subtle imaging findings while remaining robust to inter-patient variability.

The main method for renal assessment continues to be cross-sectional imaging. Computed tomography (CT) and magnetic resonance imaging (MRI) are widely used imaging modalities. These techniques provide detailed visualization of renal structures, lesions, and the surrounding tissues. Computed tomography (CT) provides high spatial resolution, enabling clinicians to detect both calcified and solid lesions [2,6]. Image interpretation is reader-dependent. Increasing imaging volumes, heterogeneous acquisition protocols, and reader-dependent interpretation may contribute to diagnostic variability, particularly in uncertain or ambiguous cases.

Deep learning has emerged as an important analytical approach for automated renal image analysis. The direct learning of spatial and textural patterns from imaging data by Convolutional Neural Networks (CNNs) enables automated renal abnormality classification. Recent hybrid and attention-based studies have also improved kidney CT classification [7,8]. These studies show that combining different feature-learning strategies can be useful. However, they give limited attention to two important issues: how the model makes its decisions and how stable it remains when image quality or clinical conditions vary. They also do not clearly show whether the same approach can work well across different datasets or diagnostic tasks. This is the main gap addressed in the present study. HMPA-CNN was designed to learn spatial and textural features in separate paths and to test that design under more diverse conditions. The initial AI applications operated with binary classification systems, which analyzed medical images between normal and abnormal states, but these systems are now used to classify images into three categories: stones, cysts, and tumors [9].

Although prior studies have reported strong performance, several barriers still limit clinical translation. Models trained on data from a single institution may not generalize well across scanners, acquisition protocols, and patient populations [2,3]. In addition, high benchmark accuracy does not necessarily reflect performance under real-world conditions characterized by noise, atypical presentations, and domain shift. Finally, limited interpretability remains a critical concern, particularly when differentiating benign from malignant tissue [5].

The existing restrictions indicate that better diagnostic results by themselves do not solve the problem. The evaluation process needs to validate results between different institutions while testing different diagnostic levels and checking performance during changes between imaging modalities to establish if learned features detect disease markers or maintain information from the training data.

This study presents a Hybrid Multi-Path Attention Convolutional Neural Network (HMPA-CNN), which uses two separate processing streams and attention mechanisms to separate spatial and textural information. The assessment of our architecture focuses on performance evaluation across five different geographic locations, three classification levels and two imaging types, which include CT and MRI. The present study investigates how AI-assisted renal imaging systems perform under different clinical situations to develop more dependable systems which doctors can use effectively.

### 1.1. Related Work and Background

Medical image classification for kidney disease automation has become a popular research topic because deep learning and computer vision technologies have advanced over the previous few years. A diverse range of architectures, including Convolutional Neural Networks, transformer-based models, and hybrid frameworks, have been proposed to address the challenges of renal pathology detection, segmentation, and classification across various imaging modalities such as computed tomography (CT) and ultrasonography. The following section reviews key studies in this field. It summarizes their main findings, experimental approaches, achieved results, and recognized research constraints.

### 1.2. Conventional CNN and Transfer Learning Studies

Firos et al. [10] presented an explainable residual learning-based transformer model called RLTNT, which worked to improve both the explainability and precision of deep learning methods for kidney disease identification. The proposed method integrates ResNet-18 for feature extraction with a Transformer-in-Transformer (TNT) architecture for classification, and was evaluated on two separate datasets comprising 12,446 and 1799 CT images, respectively. The model produced results of 99.60% accuracy, 99.58% precision, 99.60% recall and 99.59% F1-score when testing the first dataset. The article stated that the model functions best as a clinical decision-support system, which doctors should use for assistance but not as a standalone diagnostic tool. Pimpalkar et al. [11] investigated deep learning models with hyperparameter fine-tuning for the early detection and classification of kidney disorders from CT imaging. Their methodology combined transfer learning architectures such as VGG16 and ResNet50 with Watershed segmentation and Otsu thresholding techniques, and was applied to a dataset of 12,446 CT images. The research study achieved 99.96% accuracy through InceptionV3 and 100% accuracy through VGG16, but the authors noted that performance remained dependent on source data quality. Batool et al. [12] developed an explainable deep learning system using fine-tuned VGG16 with Layer-wise Relevance Propagation (LRP) to identify kidney cancer in CT images. The model achieved 98.75% accuracy on 10,000 labeled CT images, with 97.56% precision, 100% recall, and 98.76% F1-score. However, the authors acknowledged that generalizability to real-world clinical settings remained uncertain. Khan et al. [13] developed a fine-tuned deep transfer learning strategy incorporating ConvNeXt and EfficientNetV2 variants for chronic kidney disease classification. Using EfficientNetV2B0 on 12,446 CT scans, the model achieved 99.75% accuracy, 99.75% precision, 99.63% recall, and 99.75% F1-score. Despite these strong results, such transfer learning-based studies are commonly evaluated within narrowly defined benchmark settings, and their apparent robustness may not fully translate to heterogeneous clinical environments.

### 1.3. Transformer-Based and Attention-Oriented Studies

Deng et al. [14] presented a generative Vision Transformer (ViT) model, GVITKT, to address long-range dependency modeling and limited-data constraints in kidney tumor classification. The framework combined ViT-based feature extraction with GAN-based augmentation and was evaluated on 2283 tumor images and 5077 normal CT images. The model achieved 99.7% accuracy, 99.9% precision, and 99.5% F1-score, although its high computational demand remained a major limitation. Firos et al. [10] likewise incorporated transformer-based modeling with explainability components, reflecting a broader trend toward attention-enabled architectures in renal imaging. These approaches improve representational flexibility and may enhance contextual feature modeling; however, their greater architectural complexity does not, by itself, resolve persistent concerns regarding interpretability, computational scalability, and cross-site reproducibility. Moreover, explainability is often presented qualitatively, without demonstrating whether the highlighted regions correspond consistently to clinically meaningful diagnostic cues.

### 1.4. Hybrid and Ensemble Frameworks

Ghag et al. [15] developed DeepKTS-Net for kidney tumor analysis by combining fuzzy contrast enhancement with ResUNet++ and Xception architectures. Applied to 300 patients from the KiTS19 dataset, the method achieved 99.21% classification accuracy, 94.86% precision, 89.92% recall, and 90.68% F1-score. The authors identified the lack of explainable AI functionality as an important limitation. Ghosh et al. [16] proposed the STREAMLINERS system for kidney tumor detection from CT scans, integrating DenseNet121 and ResNet101 features with fuzzy logic, support vector machines, random forests, and weighted ensemble learning. Evaluated on 8260 images, the model achieved 99.2% accuracy, with 99% precision, recall, and F1-score. The study also highlighted tumor localization as an unresolved challenge. Gashmardi et al. [17] introduced a two-stage deep learning framework for automated kidney stone detection in CT images, with a U-Net for segmentation followed by CNN-based classification. Across 35,457 axial CT images, the model achieved 97.49% accuracy, 96.9% precision, 97.7% recall, and 97.3% F1-score. The authors noted that anatomical variation and poor scan quality adversely affected predictive performance. Kanchanamala et al. [18] proposed KSOMFHNet for ultrasound-based kidney stone classification, combining bilateral filtering, deep recursive residual segmentation, and SOMNet-DKN integration. On 9416 ultrasound images, the model achieved 91.984% accuracy, 90.543% recall, 90.179% precision, and 90.360% F1-score, but showed limitations in noise sensitivity and external applicability.

Collectively, hybrid and ensemble systems often achieve highly competitive results by combining complementary modules for preprocessing, segmentation, feature extraction, and classification. Nevertheless, these gains are frequently demonstrated in task-specific settings, making it difficult to determine whether performance improvements reflect genuine disease-relevant representation learning or effective adaptation to a particular dataset structure.

### 1.5. Studies with Limited Scale, Modality, or Generalizability

Svrcek et al. [19] evaluated an ultrasound image classification system combining deep learning with conventional computer vision for chronic kidney disease detection. Using 258 kidney midsagittal ultrasound images, the model achieved 82% accuracy, 71% recall, and 53% precision. The small sample size and single-institution dataset were major limitations. Al-Rasheed et al. [20] presented an optimized dense-layer deep learning model for CKD detection using a 400-record dataset, reporting 99% for accuracy, precision, recall, and F1-score. However, the restricted dataset design and fixed training regime limited the broader interpretability of these results. Caterer et al. [21] investigated feature selection strategies for the classification of kidney tissue microarrays using infrared spectral imaging. Analyzing 36,068 tissue samples from 36 tumor cores with ETC, ANOVA, and MLP, the study reported 91.3% validation accuracy and 92.7% sensitivity, while also noting the risk of patient-level overfitting.

Overall, previous studies have shown that deep learning can perform well in kidney image classification. However, several important gaps remain. Most studies test their models on only one dataset, so it is still unclear how well these methods perform across different hospitals, scanners, and patient groups. In addition, many models are developed for only one task, such as binary detection or simple multi-class classification, without testing whether the same architecture can handle more detailed diagnostic settings. Cross-modality evaluation is also limited, especially between CT and MRI. As a result, it remains uncertain whether these models learn general disease patterns or only perform well on specific datasets.

This gap motivated the present study. HMPA-CNN was developed to capture both spatial and textural features through a dual-path design, followed by attention-based refinement and feature fusion. To examine its robustness, we evaluated the model across multiple datasets, different classification settings, and both CT and MRI data. The aim was not only to achieve high accuracy but also to test whether one architecture could maintain stable performance under more diverse and clinically relevant conditions.

## 2. Materials and Methods

We assembled five independent multi-institutional imaging datasets to evaluate the performance of deep learning models for renal pathology classification across heterogeneous clinical settings. This study includes patients with various renal conditions, including cancerous and non-cancerous tumors, cysts, stones, chronic kidney disease, and healthy controls. This study included both computed tomography (CT) and magnetic resonance imaging (MRI) modalities to show the different imaging methods which occur during standard diagnostic procedures.

All data were obtained under appropriate ethical approval, and standardized anonymization procedures were applied before analysis. This study included patients from Türkiye, Bangladesh, Jordan, and Iraq, and it used public international repositories to study differences between patient groups and their imaging methods and scanner equipment. For datasets with patient-level identifiers, data partitioning was performed carefully at the patient level to avoid overlap between training and test sets. All datasets were divided using an 80:20 training-to-test split. For publicly available datasets without accessible patient-level split information, we followed the data partitioning strategies commonly used in the literature to enable fair comparison with prior studies under the same evaluation settings.

### 2.1. Dataset 1: KidneyNeXt CT Dataset

**Figure 1:**
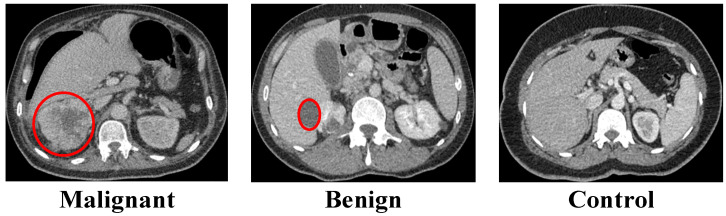
Representative axial CT images of malignant, benign, and control cases. Red circles indicate the pathological regions of interest.

The first dataset was acquired following approval from the Elazığ Fethi Sekin City Hospital Clinical Research Ethics Committee (Session No. 2025/6-20, 20 March 2025) [22]. The dataset consists of abdominal CT scans, which were collected through standard medical procedures for patient assessment. Prior to analysis, images were cropped to the renal region and fully anonymized by removing all patient identifiers. The processed images were stored in Portable Network Graphics (.png) format to facilitate standardized preprocessing. Image labeling was performed independently by experienced clinical specialists. The final class assignments resulted from consensus review, which divided the samples into three groups based on their radiological findings: malignant renal lesions, benign renal lesions, and controls who showed no signs of radiological pathology. The malignant class contained 1147 images, which displayed patients who were 60.23 ± 7.95 years old. The benign class contained 1919 images from patients who were 65.27 ± 5.22 years old and the control group consisted of 1133 images from patients who were 60.18 ± 6.25 years old. The researchers used the dataset to create their model through a process which involved dividing the data into training and independent test sets. The training subset contained 1535 benign images, together with 906 control images and 918 malignant images. The remaining samples were reserved for evaluation, including 384 benign, 227 control, and 229 malignant images. Representative axial CT examples from each diagnostic category are presented in Figure 1.

### 2.2. Dataset 2: Multi-Class Kidney CT Image Dataset

**Figure 2:**
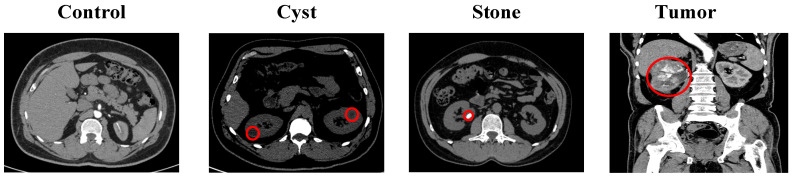
Representative CT images of kidney conditions. Red circles indicate the pathological regions of interest.

The second dataset was retrospectively curated from the Picture Archiving and Communication System (PACS) of multiple hospitals in Dhaka, Bangladesh [23]. The local radiologists who performed data acquisition and clinical diagnoses made their findings available to the public through anonymized images which they released. The dataset contains 12,446 abdominal CT images, which have been divided into four categories: normal (5077 images), cyst (3709 images), and tumor (2283 images) and stone (1377 images). The images show both contrast-enhanced and non-contrast CT scans, which were performed using standard abdominal imaging protocols to obtain axial and coronal slice images. All images were de-identified prior to release. The experimental evaluation used a pre-defined training–test split, which maintained the original class distribution. The training data consisted of 4062 normal images, 2967 cyst images, 1102 stone images and 1826 tumor images. The test data consisted of 1015 normal images, 742 cyst images, 275 stone images and 457 tumor images. Representative examples are shown in Figure 2.

### 2.3. Dataset 3: KAUH: Jordan Dataset

**Figure 3:**
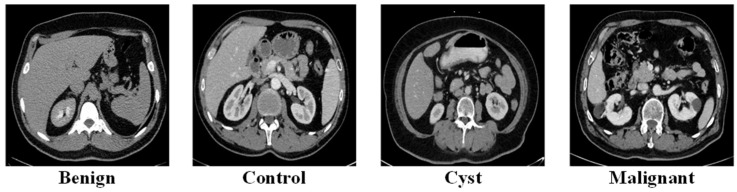
Representative axial CT images from the four-class KAUH dataset, illustrating examples of benign tumor, normal (control), cyst, and malignant renal lesions.

The third dataset originated from King Abdullah University Hospital (KAUH) in Jordan [24], through its collection of abdominal CT scans, which doctors used to diagnose various renal conditions. The researchers performed their analysis after they removed all identifying information from the images. The four-class configuration required CT image labels, which included benign, cyst, malignant and normal categories. The research used 7840 images, which scientists divided into 6272 training images and 1568 test images through an 80/20 patient-level stratified split. The class distribution maintained its stability during partitioning because this approach safeguarded against data distributional biases that existed in the information. Representative axial CT examples for each category are shown in Figure 3.

**Figure 4:**
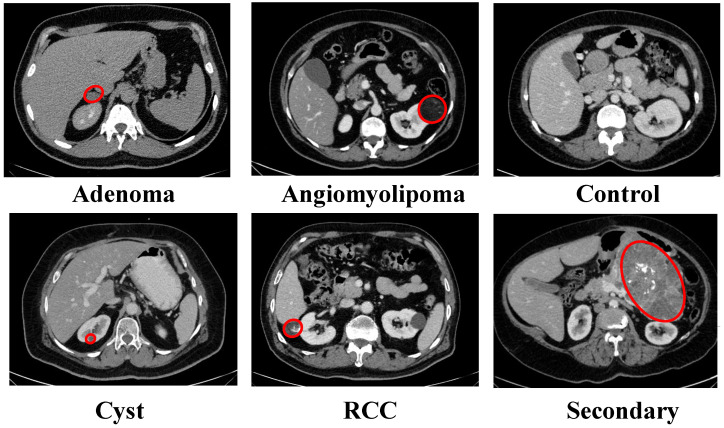
Representative axial CT images from the six-class KAUH dataset, showing adenoma, angiomyolipoma, normal (control), cyst, renal cell carcinoma (RCC), and secondary (metastatic) lesions. Red circles indicate the pathological regions of interest.

The six-class configuration included extra histopathological subtypes of benign tumors, which enabled doctors to conduct detailed diagnostic evaluations. The categories included adenoma, angiomyolipoma, cyst, renal cell carcinoma (RCC), secondary (metastatic) lesions, and normal. The six-class configuration used 7700 CT images, which were divided into training and test sets through patient-level stratified splitting at an 80/20 ratio to obtain 6160 training images and 1540 testing images. Representative axial CT examples for each class are presented in Figure 4.

The four-class configuration contains 120 distinct patients who consist of 65 males and 55 females, while their ages span from 20 to 88 years (mean age: 59.1 years). The six-class configuration contains 119 patients who consist of 64 males and 55 females with ages between 18 and 90 years old and an average age of 59.2 years. The research population in both environments consists mainly of adults between middle age and elderly who show a small difference in male to female distribution. The study designers established patient-level data partitioning to create separate training and test sets, which contained no common patients.

### 2.4. Dataset 4: Kidney Stone Detection Dataset

The fourth dataset was designed for binary kidney stone detection using abdominal CT imaging [25]. The dataset contains imaging data from patients who have renal calculi, as well as data from patients who do not show any signs of stone disease on their radiological tests. The research team obtained medical images from Sulaimani and Rania medical centers, which are located in the Kurdistan Region of Iraq, before experienced clinicians performed the image review and annotation process.

**Figure 5:**
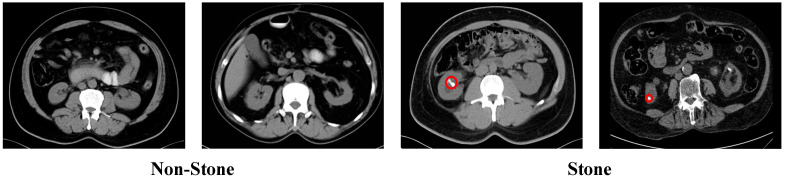
Representative axial CT images of a kidney stone. Red circles indicate the pathological regions of interest.

The dataset contains 3364 CT images, which are divided into two categories: stone and non-stone. The dataset was divided into training and test sets, and the exact class counts are reported below. The training set includes 1262 stone and 1430 non-stone images, while the test set contains 315 stone and 357 non-stone images. Representative axial CT examples are shown in Figure 5.

### 2.5. Dataset 5: Chronic Kidney Disease Dataset

**Figure 6:**
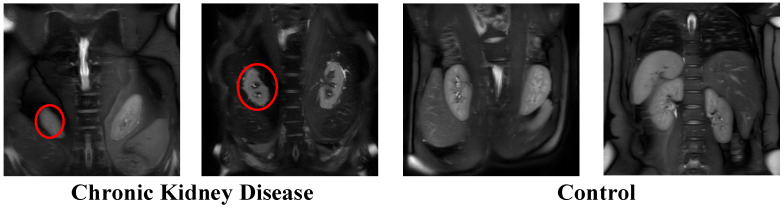
Representative Chronic Kidney Disease Dataset. Red circles indicate the pathological regions of interest.

This experiment initially used 100 T2-weighted abdominal MRI scans from a public database, including 50 CKD cases and 50 healthy controls [26]. T2-weighted imaging was selected because it provides suitable soft-tissue contrast for renal parenchymal assessment. Ten subjects underwent repeated MRI acquisitions to assess scan–rescan reproducibility. The research data contained both personal characteristics and medical records and radiology results. The CKD cohort was expanded to 1299 subjects by adding MRI data from [source]. The final dataset included 649 CKD and 650 control subjects, divided into 1039 training and 260 independent test subjects. The training set included 1039 subjects (519 CKD, 520 controls), and the independent test set comprised 260 subjects (130 CKD, 130 controls). The class distribution stayed at the same level during the entire partitioning process. Representative MRI examples are shown in Figure 6.

The research used a stepwise deep learning system to analyze five independent cohorts through their CT and MRI data for multi-class kidney disease identification. The system executed a predetermined sequence of preprocessing operations and random data augmentation methods during training to build the Hybrid Multi-Path Attention Convolutional Neural Network (HMPA-CNN), which extracted various spatial and textural features.

### 2.6. Data Preprocessing and Augmentation

**Step 1:** The proposed model used 224 × 224 pixel resizing to process all input images, which produced equal spatial dimensions between different datasets and imaging systems. The network architecture required a three-channel format, so the grayscale scans underwent channel replication to achieve compatibility. The z-score normalization method from Equation (1) was applied to standardize all pixel intensity values.

(1) $$x_{norm}=\frac{x-\mu }{\sigma }$$

Equation (1) uses x to represent the original pixel value, while μ and σ represent the mean and standard deviation values derived from the training data. The normalization process creates stable gradient updates during optimization, while it makes the system less dependent on different intensity levels between scans.

**Step 2:** The training process used stochastic geometric transformations, which MATLAB-2023’s image Data Augmenter function applied to improve model generalization and prevent overfitting. The augmentation scheme applied random rotations between ±30 degrees, it performed both horizontal and vertical shifts of 20 pixels, it applied scaling between 0.8 and 1.2 times the original size, it applied both horizontal and vertical mirror operations, and it performed shear transformations at ±15 degrees. The researchers selected these perturbations to generate acquisition variations, which would appear in real-world scenarios while preserving all necessary diagnostic information.

**Step 3:** The training process at each epoch used augmentedImageDatastore to apply dynamic transformations, which exposed the model to different image variations without requiring additional data. The inference process used only the preprocessing methods, which Step 1 defined to maintain equal evaluation conditions for untested data samples.

### 2.7. HMPA-CNN Architecture Design

**Figure 7:**
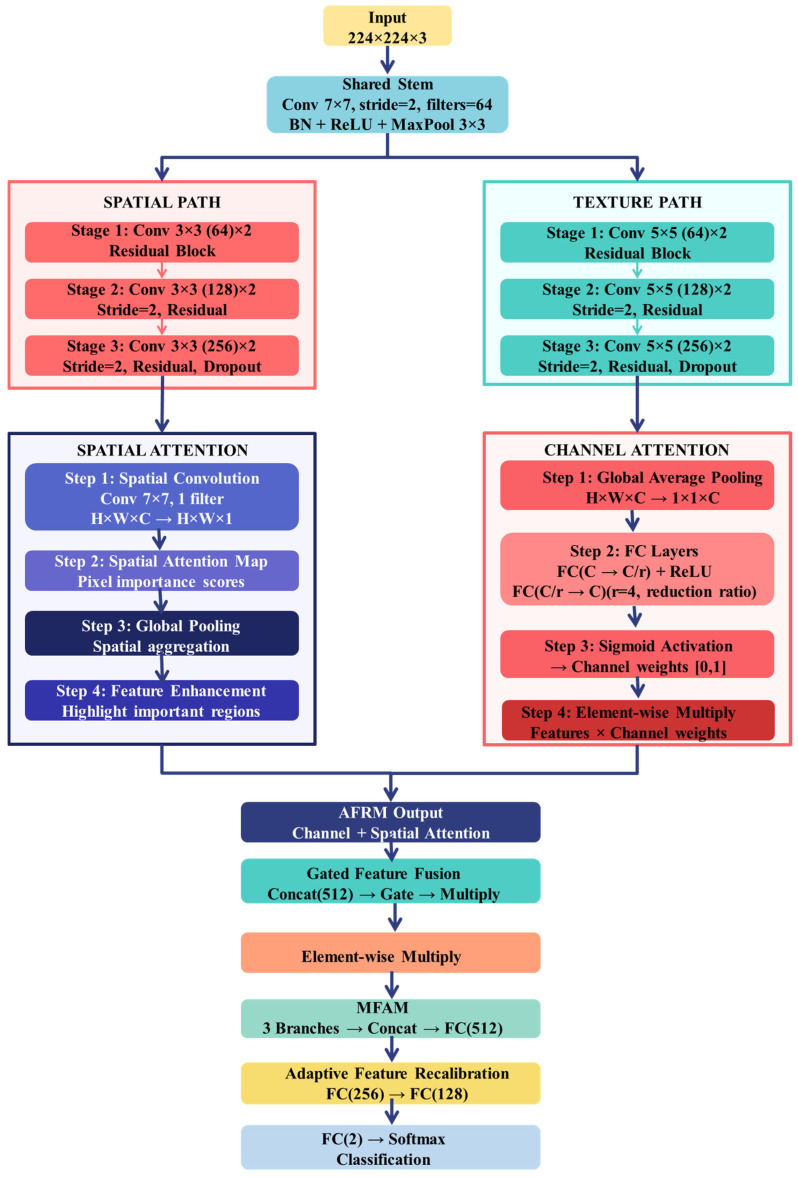
The HMPA-CNN architecture shows its dual-path structure through its attention-based feature recalibration (AFRM) and gated fusion and Multi-Branch Feature Aggregation Module (MFAM) and adaptive feature recalibration (AFR) modules, which lead to the classification output.

The Hybrid Multi-Path Attention Convolutional Neural Network (HMPA-CNN) was created to handle the different patterns found in renal images because both structural details and small texture elements help doctors make their diagnoses. This study’s contribution lies in the rigorous integration and evaluation of established deep learning components within a unified framework for heterogeneous renal imaging tasks. Its primary novelty is therefore translational rather than architectural. The dual-path structure is conceptually related to multi-scale processing strategies such as Inception, while AFRM is functionally related to channel–spatial attention mechanisms such as SE or CBAM. Our contribution lies in combining these ideas within a unified framework for heterogeneous renal imaging tasks. The system contains multiple parallel channels which extract spatial data independently from textural information before it applies attention-based feature recalibration and gated fusion. The model learns better representations through its multi-scale aggregation and adaptive refinement layers, which become active before the classification stage. The complete network design appears in Figure 7.

**Step 4:** The network accepts 224 × 224 × 3 RGB inputs, which are processed through the normalization method described in Equation (1). The stem block (layers 2–5) starts feature extraction through a 7 × 7 convolution that uses 64 filters while moving at a stride of 2 to generate feature maps which measure 112 × 112 × 64. The model applies batch normalization as a final operation, which occurs after ReLU activation. The 3 × 3 max-pooling layer with stride 2 and padding 1 reduces the representation to 56 × 56 × 64, which becomes the fundamental size for all following stages.

**Figure 8:**
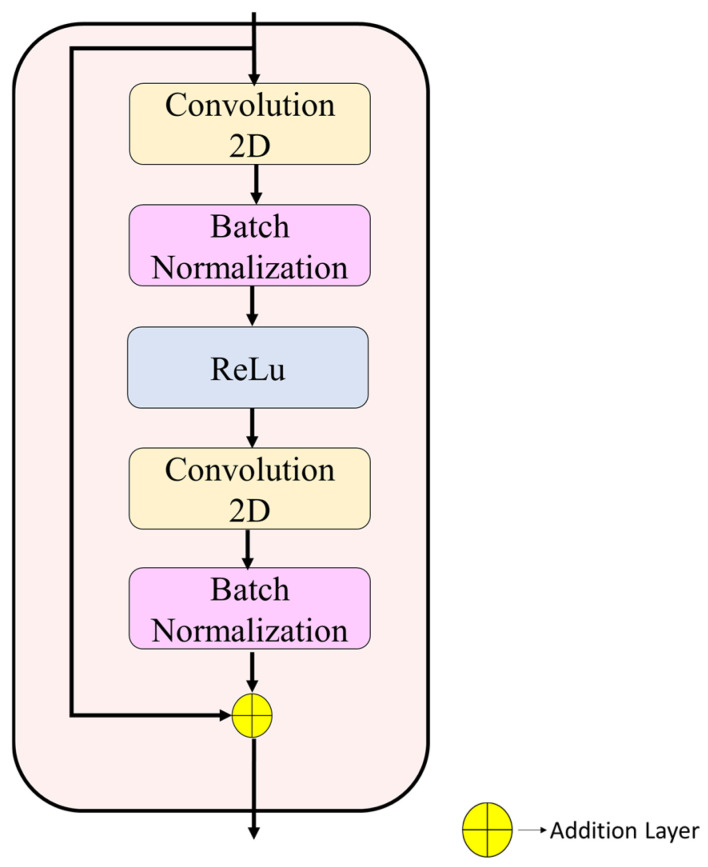
Spatial pathway Stage 1 illustrating the residual block configuration with dual convolutional layers and identity mapping.

**Figure 9:**
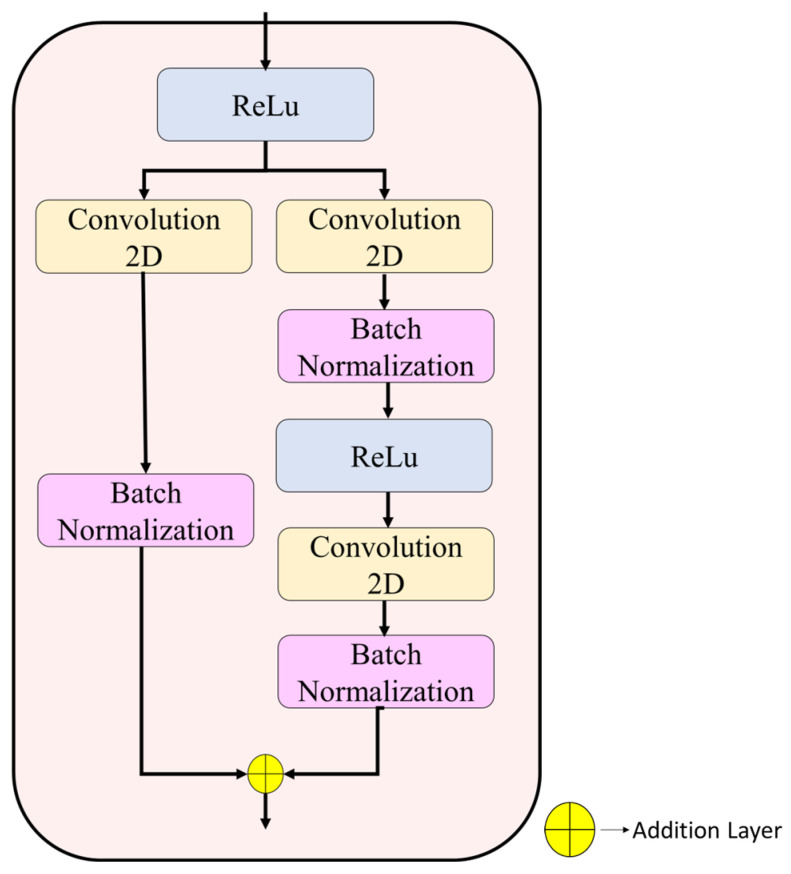
Spatial pathway Stages 2 and 3 illustrating strided downsampling, 1 × 1 transition convolutions in the skip branch, 3 × 3 convolutions in the main branch, and residual fusion.

**Step 5:** The spatial pathway (layers 6–31) uses three residual stages [27] to represent structural and boundary-related features through multiple 3 × 3 convolutional layers. The detailed configurations appear in Figure 8 and Figure 9.

Stage 1 (layers 6–12; 56 × 56 × 64): The model contains two 3 × 3 convolutional layers, which use 64 filters each and batch normalization. The block input receives a residual connection before it gets added to the output, which then passes through ReLU activation.

Stage 2 (layers 13–21; 28 × 28 × 128): Spatial downsampling is performed using a 1 × 1 convolution (stride 2, 128 filters) in the skip branch. The main branch contains a 3 × 3 convolutional layer with 128 filters and a stride of 2, which is followed by batch normalization and ReLU activation, and then a second 3 × 3 convolutional layer with 128 filters. The block ends with residual addition operations, which activate the output.

Stage 3 (layers 22–31; 14 × 14 × 256): The last spatial stage uses a residual structure to achieve 256 channel depth. The skip branch receives dimension alignment through a 1 × 1 convolution (stride 2), while the main branch consists of two 3 × 3 convolutions, which generate 256-channel outputs. A dropout layer (rate = 0.3) is applied at the end of the stage to regularize feature learning.

All convolutional layers in the spatial pathway were initialized using He initialization to maintain stable gradient propagation during training.

**Step 6:** The texture pathway (layers 45–70) operates in parallel to the spatial stream and employs 5 × 5 convolutional kernels to capture fine-grained intensity and tissue variation patterns. This design choice was based on the difference in receptive field between the two pathways. The 5 × 5 kernels were used to capture broader local intensity variation and contextual tissue patterns, whereas the 3 × 3 kernels were used to preserve finer structural detail. However, this does not imply a strict one-to-one correspondence between kernel size and feature type. Rather, the two pathways were designed to promote complementary feature learning.

Stage 1 (layers 45–51; 56 × 56 × 64): two 5 × 5 convolutional layers (64 filters) with batch normalization and residual connection.

Stage 2 (layers 52–60; 28 × 28 × 128): A 1 × 1 convolution (stride 2, 128 filters) performs dimensional alignment in the skip branch. The main branch includes two 5 × 5 convolutions (128 filters), with the first using stride 2 for spatial downsampling.

Stage 3 (layers 61–70; 14 × 14 × 256): the final stage expands to 256 channels using the same residual structure, followed by dropout (rate = 0.3).

By employing a larger receptive field, the texture pathway complements the geometry-focused representations of the spatial stream.

**Figure 10:**
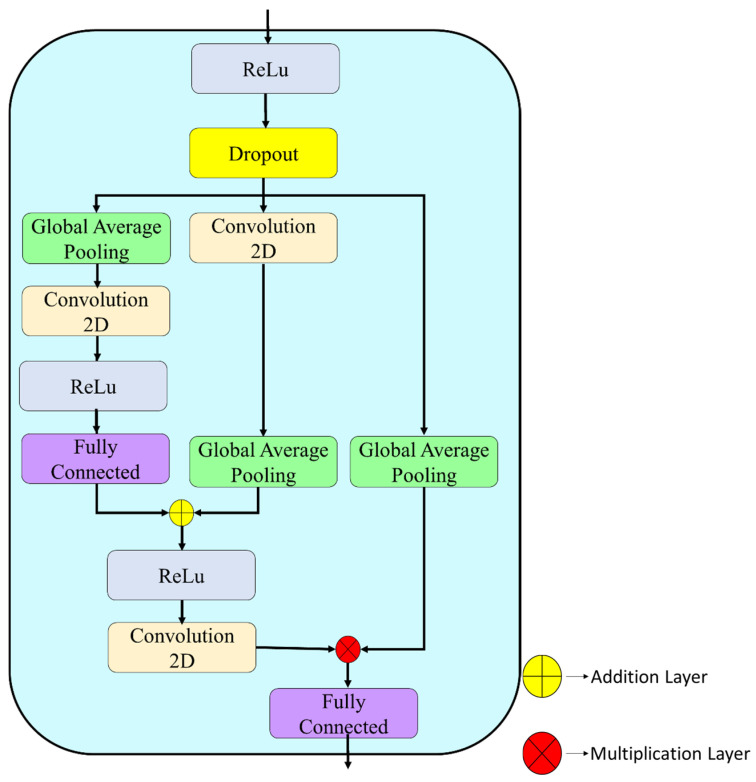
Attention-Inspired Feature Recalibration Module (AFRM) illustrating parallel channel and spatial attention branches, element-wise fusion, and generation of the recalibrated feature representation.

**Step 7:** For the spatial pathway output (14 × 14 × 256), an Attention-Inspired Feature Recalibration Module (AFRM; layers 32–44) was introduced to refine feature importance prior to fusion (Figure 10).

The channel-attention branch applies global average pooling to produce a 1 × 1 × 256 descriptor, followed by dimensionality reduction through a 1 × 1 convolution (64 filters) and ReLU activation. A fully connected layer restores the channel dimension, generating channel-wise attention weights.

In parallel, the spatial-attention branch applies a 7 × 7 convolution (single filter) to compute spatial attention maps from the 14 × 14 × 256 feature tensor, followed by global pooling to obtain spatial relevance scores.

Channel and spatial attention signals are combined via element-wise addition and activation. A subsequent 1 × 1 convolution produces 256 attention coefficients, which are applied to the original features through element-wise multiplication. The recalibrated representation is then projected through a fully connected layer (256 units) with ReLU activation, yielding an attention-weighted 256-dimensional feature vector.

**Step 8:** The same AFRM configuration (layers 71–83) was applied to the texture pathway output to maintain architectural symmetry between streams. As in the spatial branch, global average pooling was used for channel attention, while a 7 × 7 convolution generated spatial attention maps. The fused attention signals produced a 256-dimensional recalibrated feature vector for the texture pathway.

**Figure 11:**
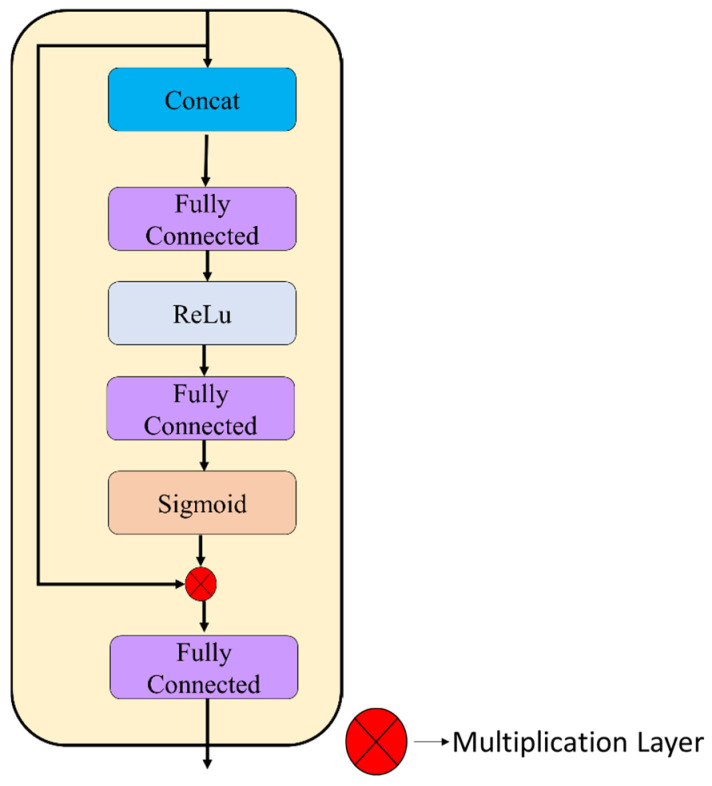
Gated feature fusion mechanism illustrating concatenation of dual-path representations, fully connected gating layers, sigmoid activation, and element-wise modulation.

**Step 9:** The attention-weighted outputs from the spatial and texture pathways (each 256-dimensional; layers 44 and 83) were combined using a learnable gating mechanism (layers 84–91), as illustrated in Figure 11.

The two vectors were first concatenated to form a 512-dimensional representation. This joint feature vector was processed through two fully connected layers (512 units each), with ReLU activation applied after the first layer. A sigmoid activation then generated element-wise gating coefficients in the range [0, 1]. The coefficients were applied through element-wise multiplication to modulate the fused feature representation. The model uses a final fully connected layer with 512 units and ReLU activation to produce a unified representation.

The sigmoid-based gating function assigns element-wise weights to the concatenated features, allowing dynamic modulation of spatial and textural contributions.

**Figure 12:**
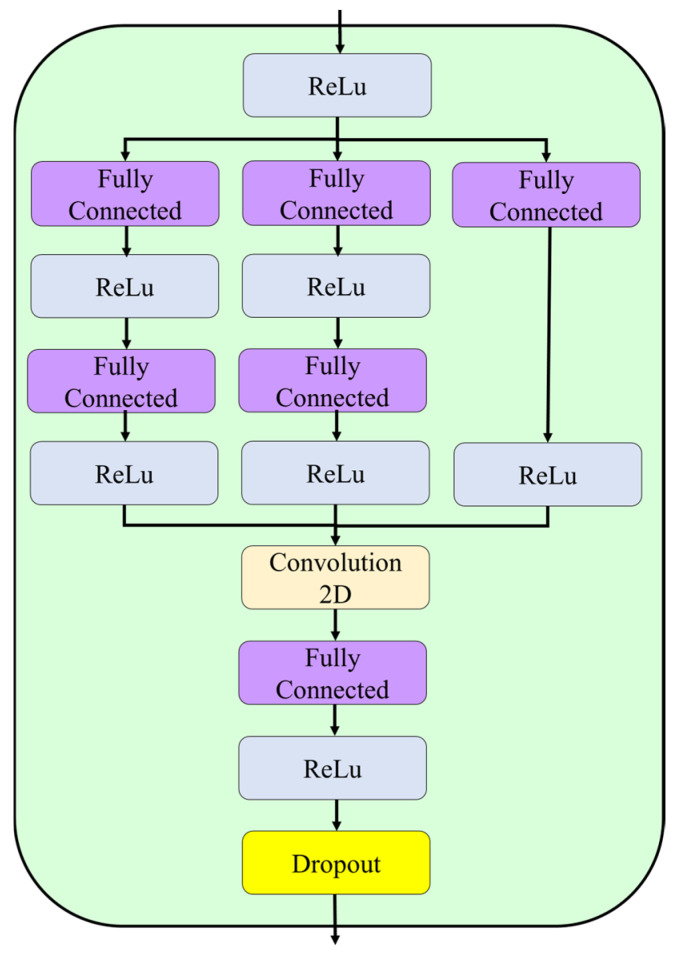
The Multi-Branch Feature Aggregation Module (MFAM) consists of three parallel transformation branches, which merge their output features before the final integration step that includes dropout regularization.

**Step 10:** The 512-dimensional representation was then processed by a Multi-Branch Feature Aggregation Module (MFAM), designed to capture complementary feature abstractions through parallel transformations with different depths (Figure 12). The module consisted of three independent branches. Branch 1 uses a single fully connected layer with 128 units (layer 92), followed by ReLU activation (layer 93), to provide a direct low-compression projection. Branch 2 uses a two-layer bottleneck structure. It included a fully connected layer with 256 units (layer 94), followed by ReLU activation (layer 95), and then a projection to 128 units (layer 96) with ReLU activation (layer 97). Branch 3 uses two consecutive fully connected layers with 128 units each (layers 98 and 100), with ReLU activation at layers 99 and 101, to learn a deeper non-linear transformation. The three 128-dimensional outputs were concatenated (layer 102) to form a 384-dimensional integrated representation. This representation was then passed through a final fully connected layer with 512 units and ReLU activation (layer 103), followed by dropout with a rate of 0.4 (layer 105) for regularization.

The network architecture contains multiple branches which process information at different levels of abstraction through separate compression routes to handle both detailed textural features and general semantic understanding.

**Step 11:** The 512-dimensional MFAM output underwent two sequential bottleneck layers (layers 106–111) for improvement. The first stage used a fully connected layer with 256 units (layer 106), which activated through ReLU (layer 107) before applying dropout with a rate of 0.3 (layer 108). The second stage extended the representation into 128 dimensions through a fully connected layer (layer 109), which used ReLU activation (layer 110) and dropout with a rate of 0.3 (layer 111). The proposed model used progressive compression to generate a 128-dimensional feature vector, which served as the final input for classification.

**Step 12:** The 128-dimensional representation was mapped to class logits through a fully connected layer (layer 112) producing C outputs, where C ∈ {2, 4, 6}, depending on the classification setting. Class probabilities were obtained using the softmax function:

(2) $$p_{i}=\frac{e^{z_{i}}}{\sum_{j=1}^{C}\;\;e^{z_{j}}}$$

where *z_i_* denotes the logit corresponding to class *i*.

Training minimized the categorical cross-entropy loss:

(3) $$L=-\sum_{i=1}^{C}\;y_{i}log\left(p_{i}\right)$$

where *y_i_* represents the ground-truth label.

The complete HMPA-CNN architecture comprises 113 layers with approximately 6.3 million learnable parameters. The information flow progresses from initial feature extraction in the stem block (56 × 56 × 64), through dual spatial and texture pathways (14 × 14 × 256 each), attention-based recalibration, gated fusion (512 dimensions), multi-scale transformation, and adaptive compression to a 128-dimensional representation prior to classification.

### 2.8. Training Protocol and Performance Evaluation

**Step 13:** Model development and training were performed in MATLAB 2023 using the Deep Learning Toolbox. The network was trained with the Adam optimizer, an initial learning rate of 1 × 10^−4^, piecewise learning-rate decay (drop factor 0.2 every 30 epochs), a maximum of 500 epochs, mini-batch size 64 (scaled by the number of GPUs when multi-GPU training was available), L2 regularization of 0.0005, and gradient clipping with a threshold of 1. Model selection was monitored on a validation subset during training, while the independent test set was used only for final performance evaluation. To quantify uncertainty in the additional stratified 10-fold evaluation, fold-wise standard deviation and the 95% confidence interval of mean accuracy were calculated.

**Step 14:** Performance was evaluated on the independent test set using the confusion matrix. For each i∈{1,…,C}, true positives ($TP_{i}$), false positives ($FP_{i}$), true negatives ($TN_{i}$), and false negatives ($FN_{i}$) were computed. From these, the following metrics were derived:

(4) $${\mathrm{Precision}}_{i}=\frac{TP_{i}}{TP_{i}+FP_{i}}$$

(5) $${\mathrm{Recall}}_{i}={\mathrm{Sensitivity}}_{i}=\frac{TP_{i}}{TP_{i}+FN_{i}}$$

(6) $${F1\text{-}\mathrm{Score}}_{i}=\frac{2\times {\mathrm{Precision}}_{i}\times {\mathrm{Recall}}_{i}}{{\mathrm{Precision}}_{i}+{\mathrm{Recall}}_{i}}$$

(7) $${\mathrm{Specificity}}_{i}=\frac{TN_{i}}{TN_{i}+FP_{i}}$$

Precision represents the proportion of correctly predicted positive samples among all positive predictions. The recall (sensitivity) metric shows the percentage of actual positive cases which the model correctly identifies. The F1-score represents the harmonic mean between precision and recall according to Equation (6), which shows their relative balance. The measure of specificity shows how well the system identifies actual negative cases because medical classification needs to avoid sending patients for unnecessary clinical tests.

**Step 15:** Overall accuracy was computed as the ratio of correctly classified samples to the total number of test samples, as defined in Equation (8).

(8) $$\mathrm{Accuracy}=\frac{\sum_{i=1}^{C}\;\;TP_{i}}{N}$$

where C is the number of classes and N is the total number of test samples. To mitigate the influence of class imbalance, macro-averaged precision, recall, and F1-score were computed by taking the unweighted mean of class-wise metrics:

(9) $$\mathrm{Macro}\text{-}\mathrm{Precision}=\frac{1}{C}\sum_{i=1}^{C}\;\;{\mathrm{Precision}}_{i}$$

(10) $$\mathrm{Macro}\text{-}\mathrm{Recall}=\frac{1}{C}\sum_{i=1}^{C}\;\;{\mathrm{Recall}}_{i}$$

(11) $$\begin{array}{c} \mathrm{Macro}\text{-}F1=\frac{1}{C}\sum_{i=1}^{C}\;\;{F1\text{-}\mathrm{Score}}_{i} \end{array}$$

(12) $$\begin{array}{c} \mathrm{Macro}\text{-}\mathrm{Specificity}=\frac{1}{C}\sum_{i=1}^{C}\;\;{Specificity}_{i} \end{array}$$

Macro-averaging assigns equal weight to each class irrespective of sample frequency, thereby preventing dominant classes from disproportionately influencing aggregate performance estimates. This formulation is particularly relevant in settings where minority diagnostic categories carry comparable clinical importance.

**Step 16:** To qualitatively assess the discriminative structure of the learned feature representations, t-distributed stochastic neighbor embedding (t-SNE) [28] was applied to the deep features extracted from multiple layers of the HMPA-CNN. Before reducing dimensions, feature vectors were standardized and then transformed into a 50-space using PCA to reduce noise and boost accuracy. Following that, t-SNE placed complex embeddings into 2D and 3D views for easier inspection. Each of the perplexity settings—5, 10, 30, 50, and 100—was tested in turn to see how much local structure changed. Training and test embeddings were analyzed separately to assess whether the learned feature structure remained stable across data partitions. In addition to visual inspection, quantitative cluster-separation metrics were calculated, including inter-class distance, intra-class compactness, the Davies–Bouldin index, and silhouette scores. On top of that, how puzzling things get depending on perplexity showed up in simpler indexes built when flattening dimensions into two dimensions. The t-SNE plots were used only for qualitative visualization of feature separability. Because t-SNE is sensitive to parameter settings, especially perplexity, these embeddings should be interpreted with caution and not as a definitive representation of the original high-dimensional feature space. To reduce this limitation, multiple perplexity values were tested and the overall clustering pattern was checked for consistency.

Inference efficiency was also assessed on the KidneyNeXt test set. The proposed model showed a mean inference time of 11.14 ms per image (median, 12.10 ms; range, 8.13–16.64 ms), corresponding to a throughput of 89.78 images/s under the tested hardware setting.

## 3. Results

The HMPA-CNN architecture was evaluated using five separate datasets, which included different renal diseases and various medical imaging types and multiple healthcare facilities. The evaluation process included three different classification tasks, which used binary labels, four-class labels and six-class labels to test different diagnostic precision levels. The system used five performance metrics to evaluate its results, which consisted of precision, recall, specificity, F1-score and overall accuracy. The analysis of confusion matrices helped researchers understand how the model performed on different classes through error pattern evaluation, and receiver operating characteristic (ROC) curves measured the model’s ability to discriminate between classes. In addition, t-distributed stochastic neighbor embedding (t-SNE) was applied to the learned feature representations to visualize class separability in the embedding space.

### 3.1. Dataset 1: KidneyNeXt CT Dataset

**Figure 13:**
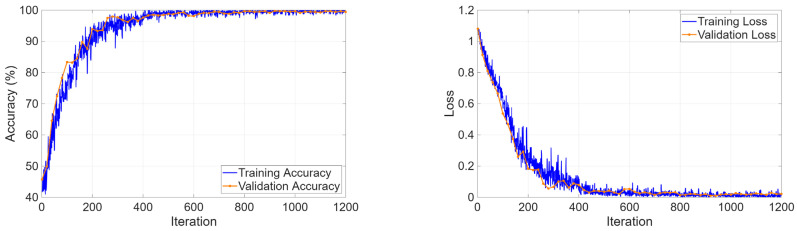
Training and validation curves for the KidneyNeXt dataset.

The three-class classification task on the KidneyNeXt dataset required the model to distinguish between benign tumors and malignant lesions and normal controls. The training curves in Figure 13 showed that the optimization process operated steadily because accuracy reached 99% during the first training epochs and maintained this level throughout the entire training period. The loss function demonstrated continuous improvement through its training and validation performance curves, which showed no major differences between each other.

**Figure 14:**
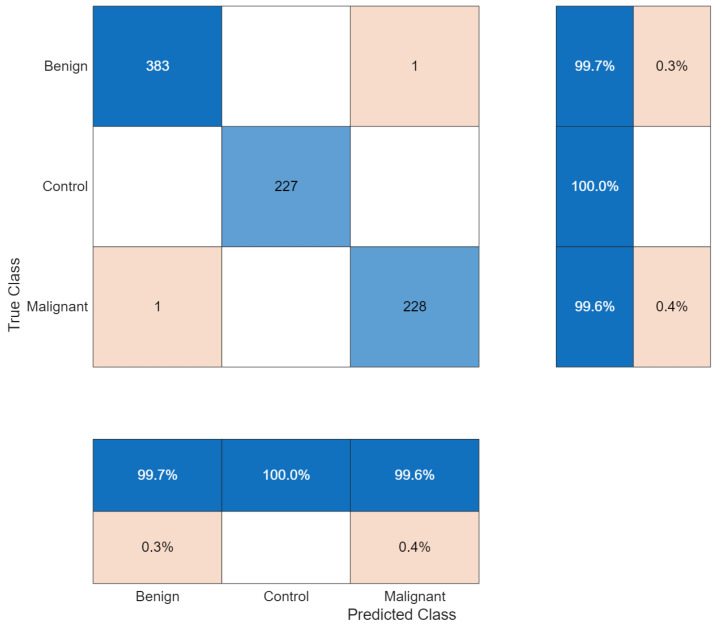
Confusion matrix for three-class classification.

The confusion matrix showed two misclassifications among 840 test samples. The control class was classified without error (227/227), while one benign case and one malignant case were misclassified. The three-class confusion matrix is shown in Figure 14.

Class-wise performance metrics are summarized in Table 1. The system achieved 99.74% precision and recall for benign lesions and 99.56% precision and recall for malignant lesions, but the control group reached 100% precision and recall. Macro-averaged precision and recall were 99.77%, and overall accuracy reached 99.76%.

**Figure 15:**
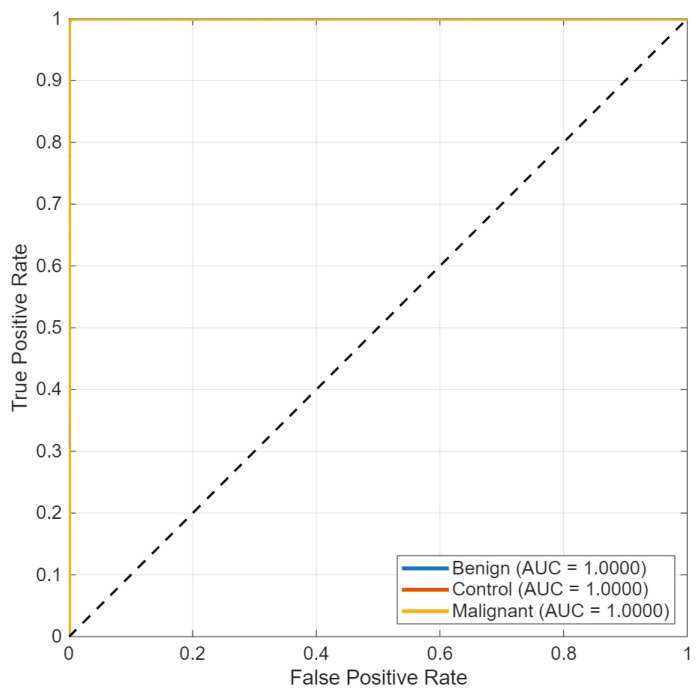
Multi-class ROC curves for the KidneyNeXt dataset.

Receiver operating characteristic analysis (Figure 15) yielded area under the curve (AUC) values above 0.99 for all three classes. The ROC curves from micro- and macro-averaging methods showed similar behavior as they moved toward the top-left corner of the plot, which demonstrated their ability to make accurate predictions at different threshold settings.

**Figure 16:**
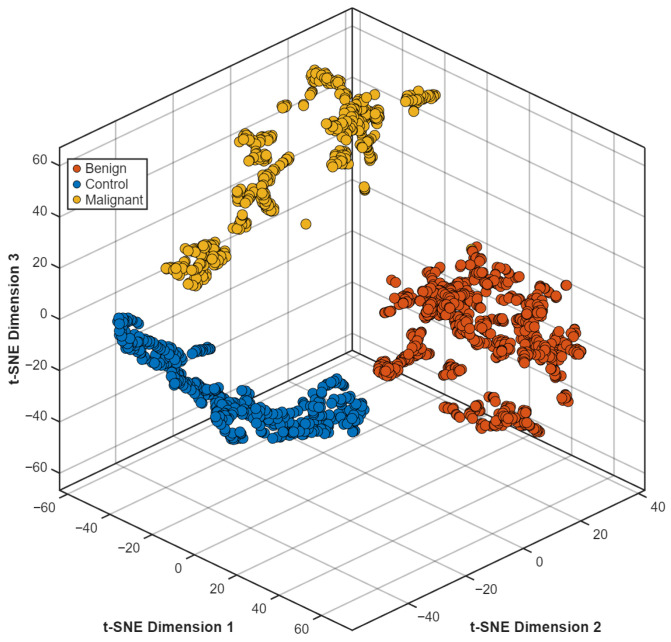
t-SNE projection of the learned feature embeddings.

The t-SNE visualization of the learned feature space (Figure 16) showed distinct clusters which separated benign from malignant and control samples. The diagnostic groups showed equal representation through their features because these features maintained their distinct patterns across all class boundaries.

#### Comparisons with Professionals

To provide an initial clinical reference point, we performed a small comparison between the proposed model and human readers using the KidneyNeXt CT dataset. A total of 45 axial CT images were selected, including 15 benign cases, 15 control cases, and 15 malignant cases. This comparison is exploratory and not statistically powered. These images were presented through Google Forms to 10 radiology specialists, who were asked to assign each image to one of the three diagnostic categories. In this evaluation, the proposed HMPA-CNN classified 44 of 45 images correctly, with only one misclassification, corresponding to an overall accuracy of 97.78%. Across the same image set, the radiology specialists made five errors in total, including two benign cases, one control case, and two malignant cases, corresponding to an overall accuracy of 88.89%. These findings suggest that the proposed model may provide useful support in renal image classification under controlled conditions.

### 3.2. Dataset 2: Multi-Class Kidney CT Image Dataset

**Figure 17:**
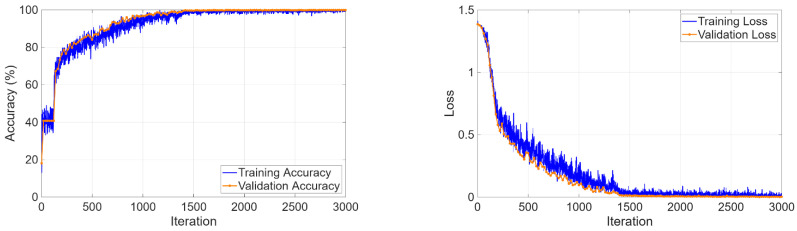
Learning curves for four-class classification.

The four-class classification task required simultaneous discrimination among cyst, stone, tumor, and normal tissue. The training curves in Figure 17 showed rapid learning progress because validation accuracy reached 99.98% during the initial epochs and loss values achieved their minimum value.

**Figure 18:**
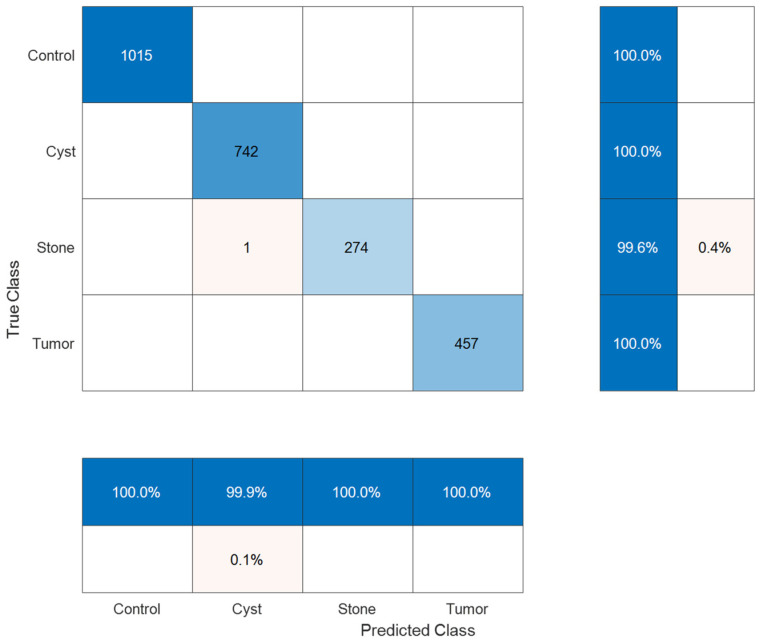
Confusion matrix for four-class classification.

The confusion matrix (Figure 18) showed that two of 2489 test samples were misclassified. The cyst class achieved 100% recall with one false positive. The stone detection process achieved 99.80% recall accuracy through a single incorrect detection, which resulted in a false negative result. Control and tumor classes were classified without observed error in the test set.

The results from performance testing for all classes appear in Table 2. The system achieved precision between 99.87% and 100%, while it obtained recall values between 99.64% and 100%. The model achieved a macro-average precision of 99.97%, recall of 99.91%, specificity of 99.99%, and F1-score of 99.94%, with an overall accuracy of 99.96%.

**Figure 19:**
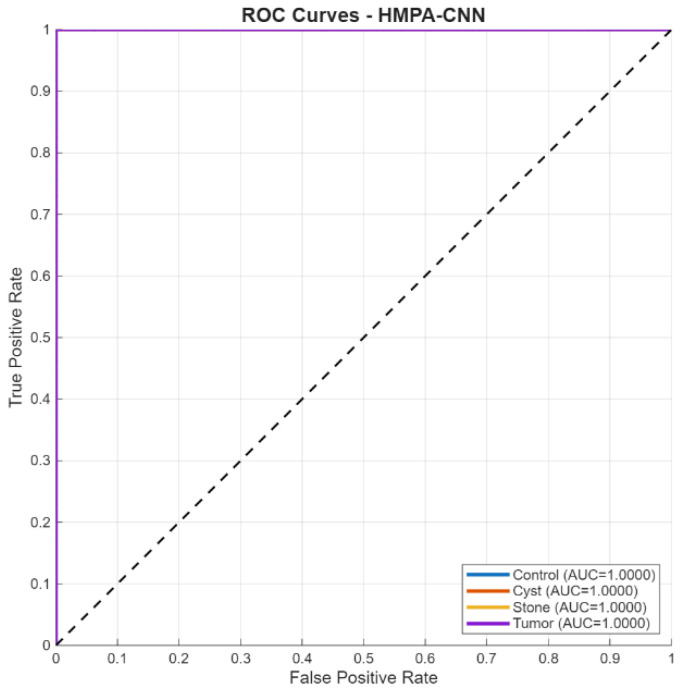
ROC curves for four-class classification.

Receiver operating characteristic analysis (Figure 19) yielded AUC values above 0.99 for all classes, indicating strong separability across decision thresholds.

**Figure 20:**
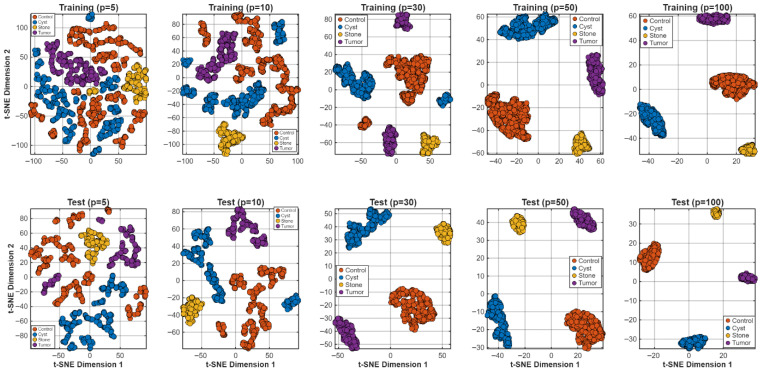
Class-wise t-SNE visualization.

**Figure 21:**
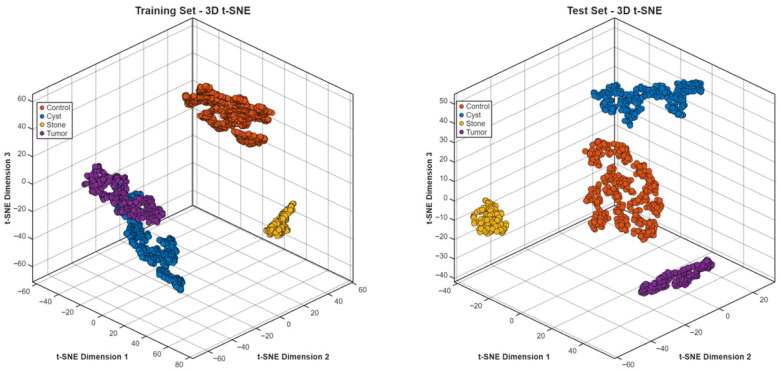
Unified t-SNE embedding of all four classes.

Feature space structure was examined using t-SNE projections (Figure 20 and Figure 21). The two-dimensional class-wise embeddings displayed tight clustering patterns, which maintained small areas of overlap between their boundaries. The global embedding showed that the learned representation space contained distinct information between control, cyst, stone and tumor groups.

The present study investigated how training sample sizes affect representation stability through t-SNE analysis of training data subsets, which grew from p = 5, 10, 30, 50, and 100 samples per class. The assessment of smaller datasets showed that different categories which have similar morphological characteristics display specific degrees of similarity. The cluster compactness increased while inter-class separation became more defined when the number of samples increased. The training and test embeddings showed identical geometric patterns, which indicates that the data partitions received equivalent representation.

### 3.3. Dataset 3: KAUH: Jordan Dataset

**Figure 22:**
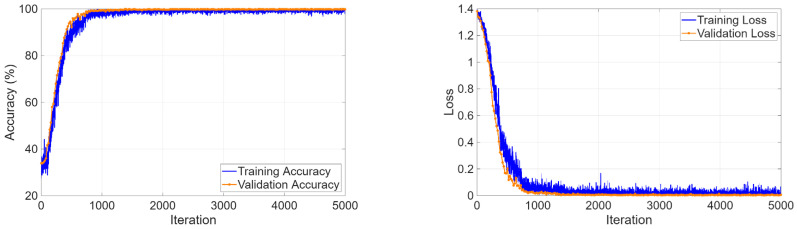
Training curves for four-class cross-institutional evaluation.

The Jordan cohort functioned as an external validation group, which used different acquisition methods and scanner equipment and contained patients with distinct characteristics. The model achieved a validation accuracy of 99.74% in the four-class setting, which included benign lesions, control cases, cysts, and malignant lesions. The model maintained stability throughout training because its training and validation performance tracks showed no major changes between them. The training curves for the four-class cross-institutional evaluation are shown in Figure 22.

**Figure 23:**
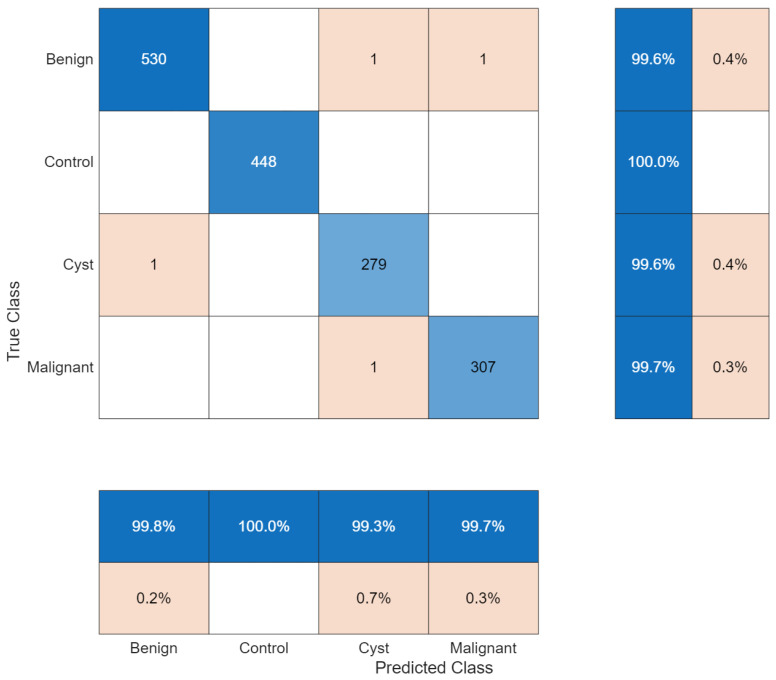
Confusion matrix for the Jordan four-class dataset.

Among 1568 test samples, four were misclassified (Figure 23). Errors were primarily confined to limited confusion between benign lesions and cysts or malignant tumors. This study performed error-free classification of control cases. The system identifies different groups through its institutions, which operate under distinct regulatory systems.

The class-wise metrics (Table 3) presented precision values which ranged from 99.29% to 100% and recall values which ranged from 99.62% to 100%. Macro-averaged precision and recall were 99.69% and 99.74%, respectively, with specificity of 99.92%.

**Figure 24:**
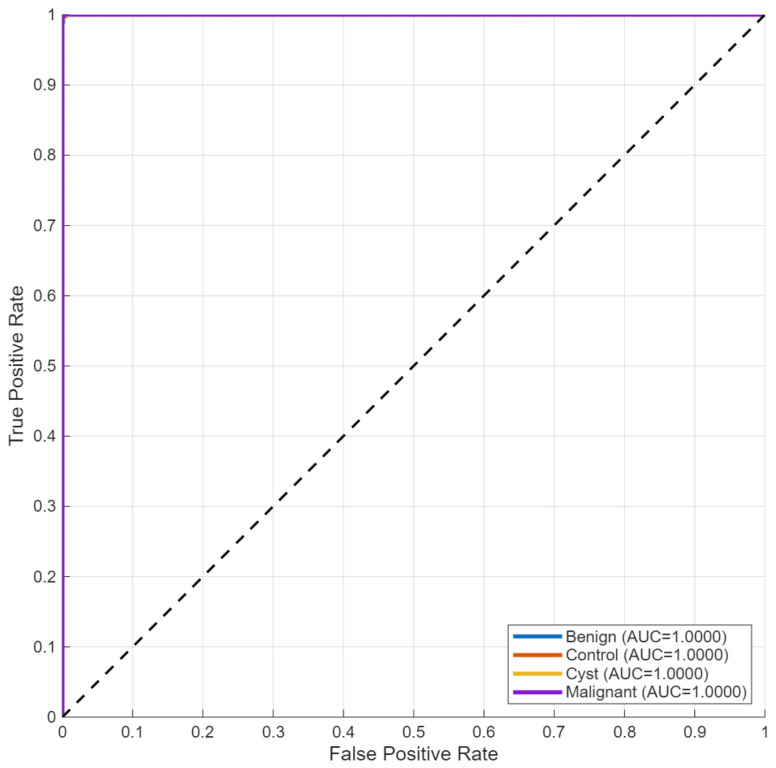
ROC curves for the Jordan four-class configuration.

Receiver operating characteristic analysis (Figure 24) yielded AUC values above 0.99 across all classes, supporting consistent separability across decision thresholds.

**Figure 25:**
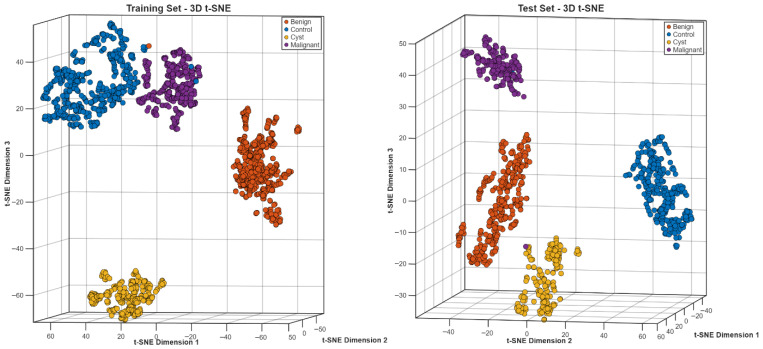
t-SNE embedding for the Jordan dataset.

The t-SNE projections in three dimensions (Figure 25) showed specific clustering patterns, which matched the different diagnostic groups. The training and test embeddings maintained identical spatial patterns, which demonstrated that the feature representation stayed unchanged between different data segments.

**Figure 26:**
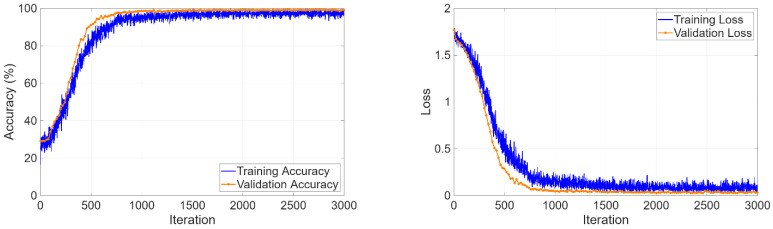
Training curves for six-class tumor subtype classification.

The six-class system introduced better discrimination between adenoma, angiomyolipoma, control, cyst, renal cell carcinoma (RCC) and secondary malignancy. The task needed exact subtype identification, which produced an overall accuracy rate of 96.36% (Figure 26).

**Figure 27:**
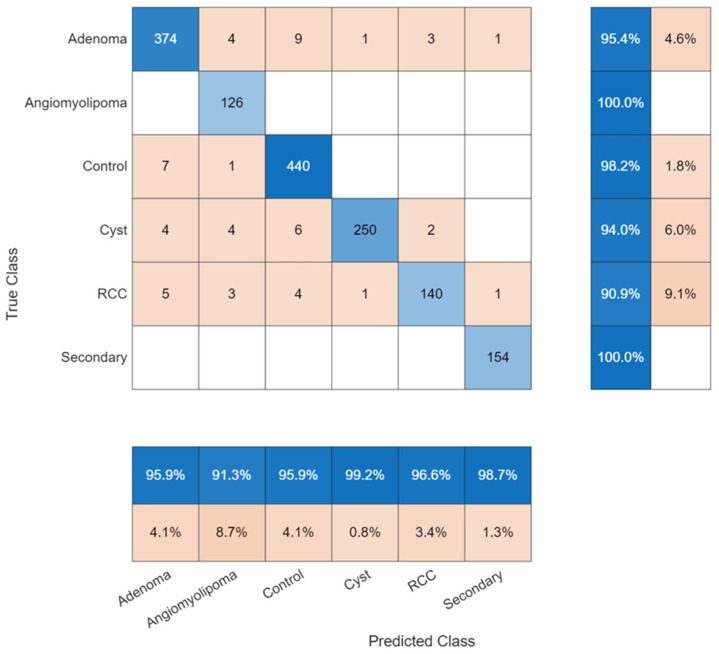
Confusion matrix for six-class classification.

Most misclassifications occurred between morphologically similar subtypes which shared morphological characteristics (Figure 27). RCC achieved a recall of 90.91%, indicating greater confusion with other subtypes than the remaining classes. The proposed model achieved 95.41% adenoma detection accuracy and 100% angiomyolipoma detection accuracy. The system achieved 98.72% precision and 100% recall for its identification of secondary malignancies.

The model achieved 96.26% precision and 96.42% recall at the macro level according to Table 4. The experimental data showed better performance than standard classification experiments, yet the system maintained its ability to distinguish between classes when it received more diagnostic information.

**Figure 28:**
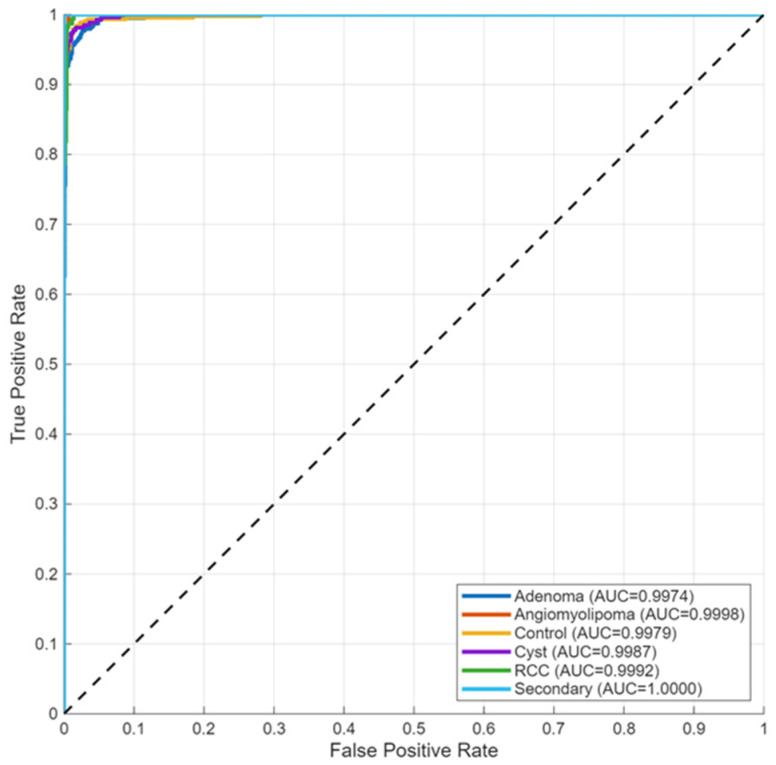
ROC curves for six-class tumor classification.

**Figure 29:**
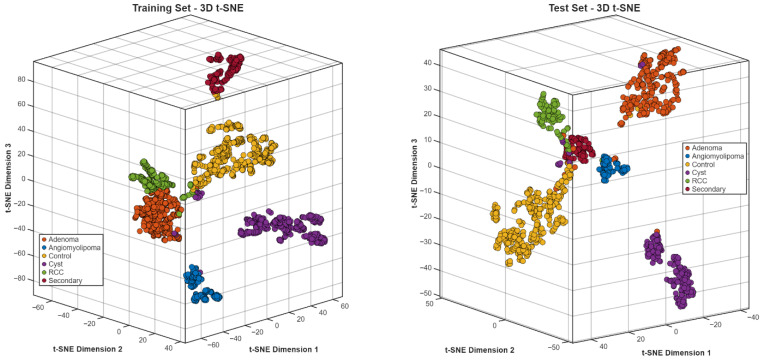
t-SNE embedding for six-class subtype differentiation.

ROC analysis (Figure 28) yielded AUC values above 0.95 for all subtypes. Embedding visualizations (Figure 29) revealed subtype-specific clustering with varying degrees of compactness; RCC clusters showed greater dispersion relative to other categories, consistent with subtype variability.

The three-dimensional t-SNE projection for the six-class configuration (Figure 29) shows subtype-specific clustering within the learned feature space. The different tissue types, including adenoma, angiomyolipoma, control, cyst, RCC and secondary malignancy, exist as separate areas which show minimal shared territory between their distinct morphological characteristics. The cluster compactness shows different levels of variation between subtypes because RCC clusters tend to spread out more than the other classes. The training and test embeddings show identical spatial patterns, which demonstrates that the data representation stays consistent between different data segments.

### 3.4. Dataset 4: Kidney Stone Detection Dataset

**Figure 30:**
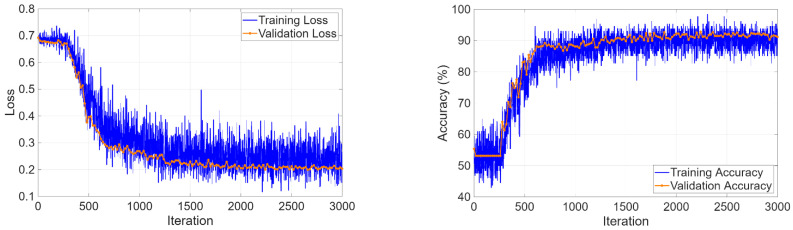
Learning curves for kidney stone detection.

The kidney stone dataset presented a binary detection task, which required users to identify stone-positive or stone-negative CT scans. The task required the model to detect small high-density structures which appear inside the kidney tissue instead of working on mass identification tasks. The model reached 91.96% validation accuracy when it converged (Figure 30), while it decreased loss values throughout the training process.

**Figure 31:**
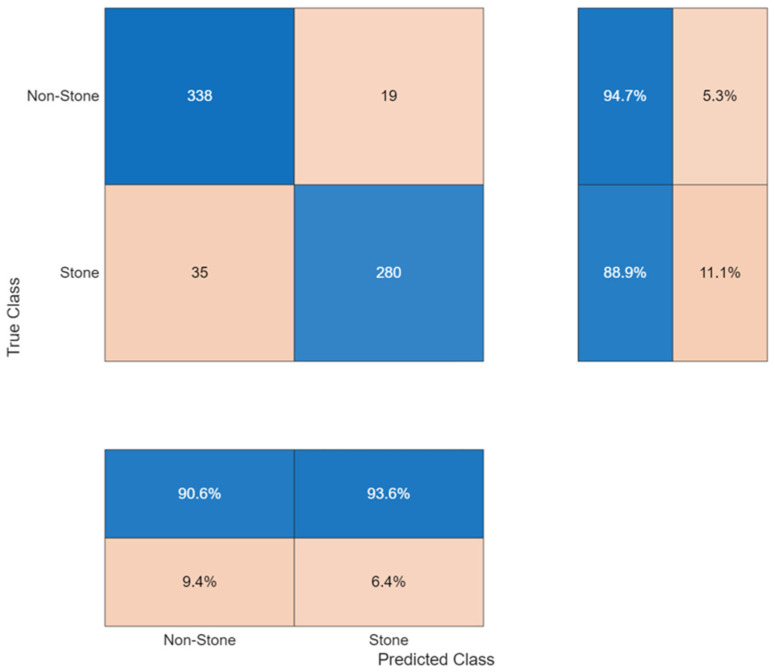
Confusion matrix for binary stone detection.

The confusion matrix (Figure 31) presented errors which were balanced between different categories, yet the errors did not occur at equal rates. The non-stone cases produced 94.68% recall accuracy through 14 incorrect positive predictions. The stone-positive cases produced an 88.89% recall rate, which resulted in 12 incorrect missed detections.

The performance data appears in Table 5. Precision was 90.62% for non-stone and 93.65% for stone cases. The system achieved 92.13% precision and 91.78% recall at the macro level while maintaining 91.96% overall accuracy. To further examine the stability of the kidney stone detection results, a stratified 10-fold evaluation was performed on the test cohort. Fold-wise accuracy ranged from 85.29% to 95.59%, with a mean accuracy of 91.97% ± 3.05% and a 95% confidence interval of 89.78–94.15%. The corresponding mean precision, recall, and F1-score were 92.22%, 91.79%, and 91.91%, respectively. These findings indicate moderate fold-to-fold variability, supporting the interpretation that stone detection is more sensitive to sample-level heterogeneity than the renal mass classification tasks.

**Figure 32:**
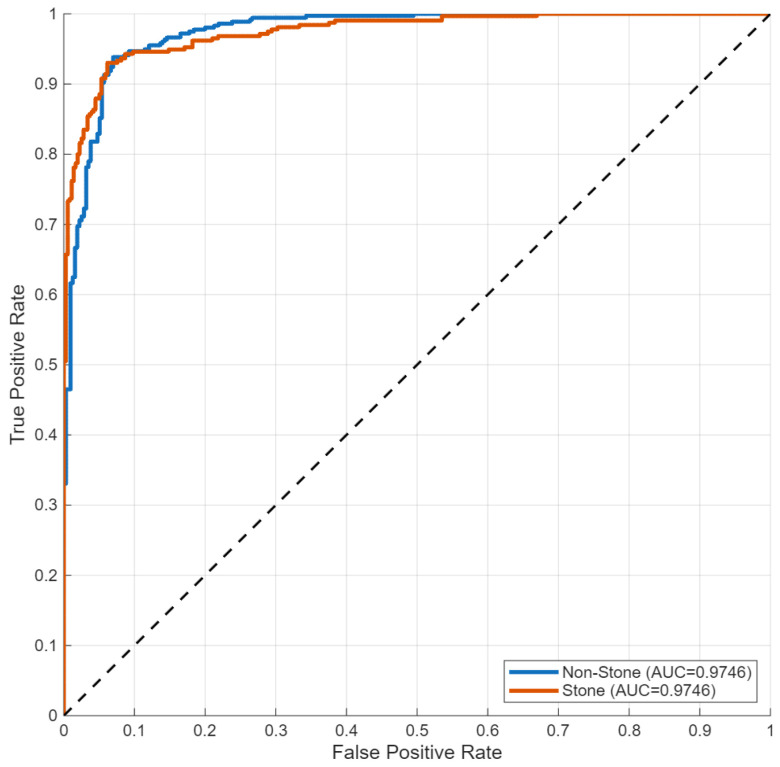
ROC curve for binary stone detection.

ROC analysis (Figure 32) yielded an AUC of 0.92, indicating consistent discrimination, although lower than values observed in tumor classification tasks.

**Figure 33:**
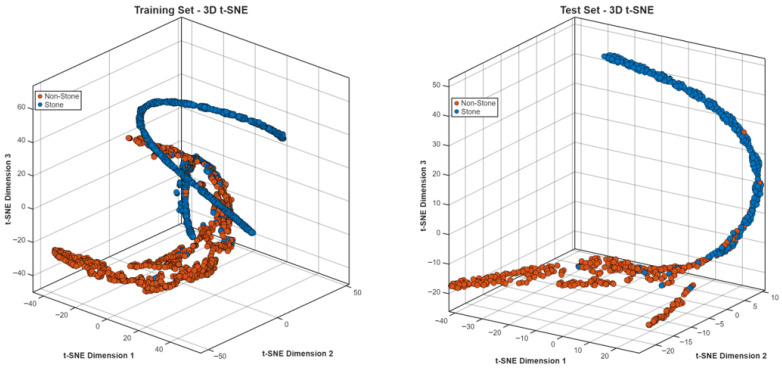
t-SNE embedding for stone and non-stone cases.

The stone and non-stone representations in Figure 33 show some degree of overlap through t-SNE projections, which display manifolds that extend rather than form distinct clusters. The classes in this task showed stronger geometric closeness than what was seen in tumor classification problems, which resulted in decreased classification accuracy.

### 3.5. Dataset 5: Chronic Kidney Disease Dataset

**Figure 34:**
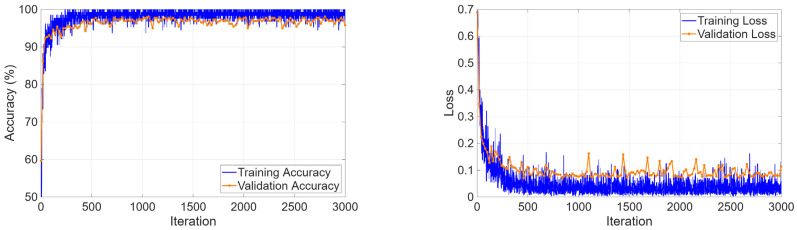
Training curves for MRI-based CKD classification.

The CKD dataset presented a cross-modality evaluation, which moved from CT-based mass detection to T2-weighted MRI assessment of parenchymal disease. The model converged to 93.85% validation accuracy (Figure 34), with stable training and validation trajectories.

**Figure 35:**
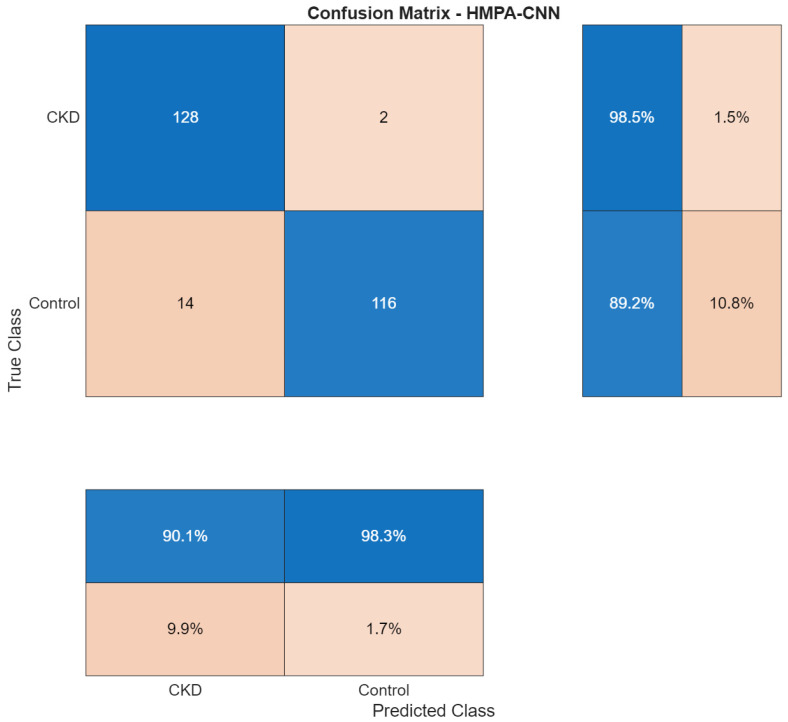
Confusion matrix for CKD classification.

The confusion matrix (Figure 35) showed that the model produced different error rates when predicting between the two classes. The system achieved 98.46% recall for CKD cases, but it incorrectly identified two patients as non-CKD cases. The control cases showed 89.23% recall, which resulted in 14 incorrect positive predictions.

The performance data appears in Table 6. The system achieved 90.14% precision for CKD detection and 98.46% recall for CKD detection, while control classification produced 98.31% precision and 89.23% recall. The model achieved 94.22% precision, 93.85% recall and 93.85% specificity in macro-averaged evaluation while showing 93.85% overall accuracy.

**Figure 36:**
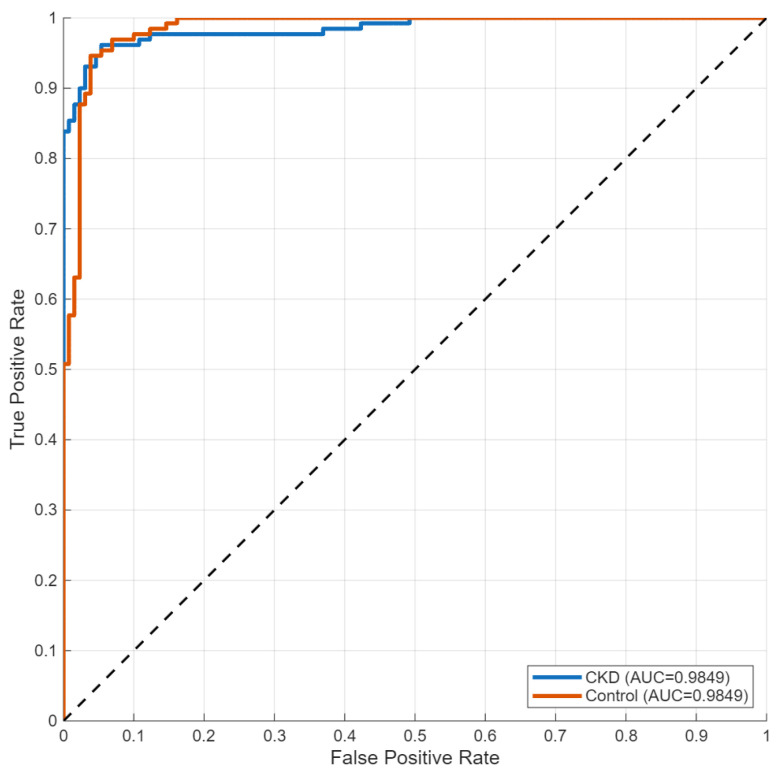
ROC curve for CKD classification.

ROC analysis (Figure 36) yielded an AUC of 0.94, indicating consistent discrimination in the MRI cohort.

**Figure 37:**
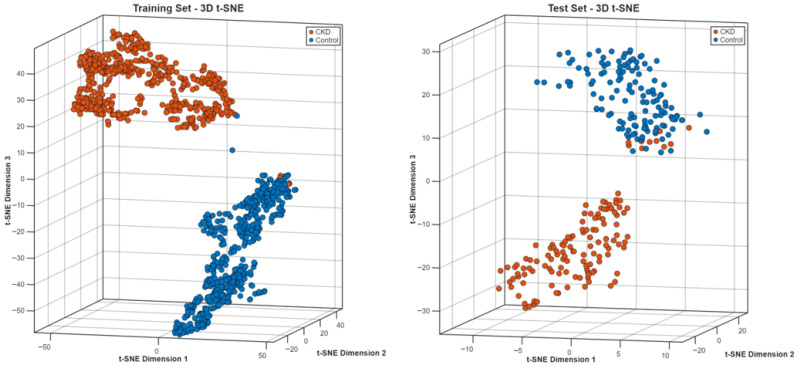
t-SNE embedding for MRI-based CKD dataset.

The t-SNE visualization (Figure 37) showed that the CKD and control groups created separate clusters, which stayed different from each other between training and test datasets. The cluster boundaries in this study showed higher variability than CT-based tumor classification tasks because the method produced a moderate decrease in classification performance.

### 3.6. Grad-CAM Analysis for KidneyNeXt CT Dataset

**Figure 38:**
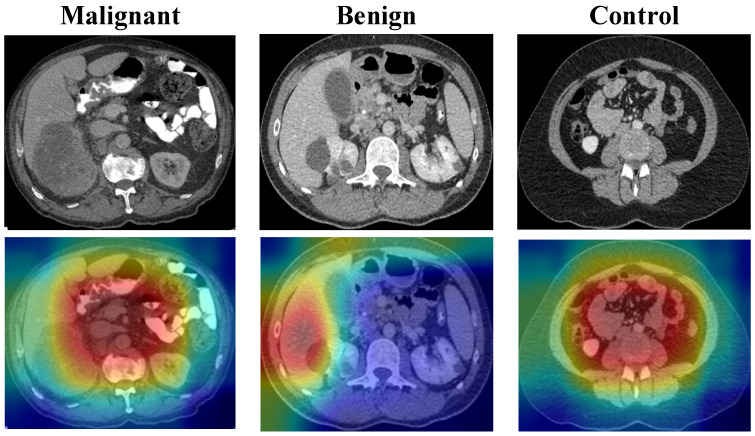
Representative Grad-CAM visualizations for malignant, benign, and control cases from the KidneyNeXt CT dataset. The first row shows the original CT images, and the second row shows the corresponding Grad-CAM heatmaps generated by the proposed HMPA-CNN.

To further examine the visual behavior of the proposed model, Grad-CAM [29] analysis was performed on representative samples from the KidneyNeXt CT dataset, including malignant, benign, and control cases (Figure 38). The resulting heatmaps suggested that the model assigned higher saliency to renal regions rather than to peripheral background structures. In the malignant case, the activation map was concentrated over the lesion-bearing renal area. In the benign case, the model likewise highlighted the renal mass region. In the control image, attention remained centered on the renal and adjacent abdominal region without a focal lesion-specific hotspot.

Expert radiological review of the representative Grad-CAM outputs by radiology specialist Dr. Gülay Maçin indicated that the malignant case exhibited a broader and more heterogeneous activation pattern centered on the lesion-bearing renal parenchyma, with extension into the adjacent abnormal-appearing tissue, a distribution compatible with the more complex and potentially infiltrative imaging phenotype of malignant renal masses on CT. In contrast, the benign case demonstrated a more spatially confined and relatively homogeneous activation pattern, predominantly localized to the contours of the renal mass without comparable extension into the surrounding parenchyma. This qualitative distinction is consistent with the more circumscribed radiological appearance commonly associated with benign renal lesions.

These visual findings provide preliminary qualitative support that the proposed architecture may be capturing clinically meaningful image patterns rather than relying solely on abstract class separation. At the same time, Grad-CAM should be interpreted as supportive evidence of model attention rather than a direct explanation of decision logic, and these observations do not constitute lesion-level validation or definitive proof of diagnostic reasoning.

**Figure 39:**
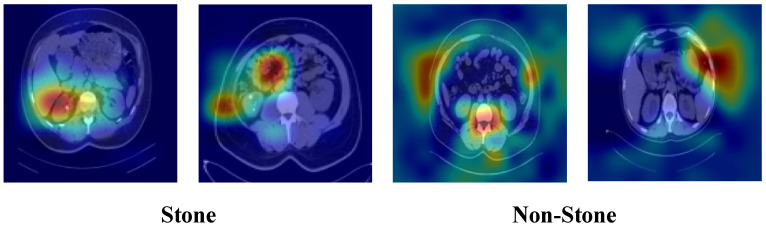
Grad-CAM visualizations for representative stone and non-stone CT images.

To further examine the reduced performance observed in kidney stone detection, Grad-CAM analysis was also performed on representative stone and non-stone CT images (Figure 39). As shown in Figure 39, the model focused on focal renal regions in stone-positive images, whereas attention in non-stone images was more diffuse and occasionally extended beyond the kidney region.

## 4. Discussion

Artificial intelligence-based systems have made major progress in kidney disease classification since 2013, as many studies have reported accuracies exceeding 95%, with several benchmark datasets yielding near-perfect results. The present study investigated a dual-path attention-based convolutional system through testing with five separate datasets, which included tumor identification, kidney stone detection and chronic kidney disease evaluation from CT and MRI images. The model demonstrated consistently high empirical performance across multiple renal imaging tasks, with the highest accuracies observed in CT-based tumor classification settings. However, performance decreased in kidney stone detection and in cross-modality CKD classification, indicating that task complexity, lesion characteristics, and modality shift remain important sources of performance variability. This study aims to create methods which generate dependable results from various data collections instead of maximizing test accuracy performance. The architecture produced equivalent results when tested on three separate CT datasets, which included tests between different medical facilities, and it maintained its ability to distinguish between different conditions during MRI-based parenchymal disease evaluation.

Performance variability across datasets was further examined as an indirect indicator of domain shift. The three CT-based renal mass datasets showed highly consistent accuracy values, ranging from 99.74% to 99.96%, suggesting limited performance degradation across institutional CT cohorts. In contrast, accuracy decreased to 96.36% for six-class tumor subtyping, 93.85% for MRI-based CKD classification, and 91.96% for kidney stone detection. This pattern suggests that performance variability was driven more by diagnostic granularity, lesion scale, and imaging modality than by institutional differences within CT-based renal mass classification. The six-class tumor subtyping and stone detection results showed a decrease because the model responds based on both the complexity of tasks and the specific characteristics of lesions. Although formulated as a binary task, kidney stone detection is intrinsically challenging because stones often appear as small, focal hyperdense structures occupying a limited portion of the CT slice. From a clinical perspective, false-negative stone predictions may delay further evaluation and management of obstructive disease, whereas false-positive predictions may increase unnecessary follow-up imaging or clinical workload. Their visibility may be affected by slice thickness, partial-volume effects, adjacent calcifications, and local image noise. In contrast, tumor and cyst classes generally provide broader anatomical and parenchymal cues, which may explain the comparatively higher performance in multi-class renal mass classification. The performance trends show detailed performance data, which goes beyond the basic headline accuracy metrics.

Table 7 shows that several previous studies have achieved accuracy values above 99% on the 12,446-image CT benchmark. CT benchmark achieved high accuracy through three different approaches, which included CNNs [11], ensemble models [30] and attention-augmented architectures [31]. PCSANet [31] represents an attention-based design with pyramid channel and spatial attention, whereas SpinalCNN [32] reflects a hybrid strategy that combines convolutional learning with a SpinalNet-based classifier. The research shows that CT classification with four classes at a coarse level achieves high accuracy across different architectural systems. Under such conditions, incremental numerical improvements provide limited differentiation between models. However, differences in evaluation protocols limit direct comparisons across studies. Several prior works focus primarily on single-dataset validation [11,30,33,34], whereas cross-dataset performance has shown variability in some studies (e.g., performance decline reported by Khan et al. [35] under cross-cohort testing). The research produced stable results through three separate CT dataset tests, which produced accuracy rates between 99.74% and 99.98%. Research on this topic remains limited, as relatively few studies have moved beyond the typical four-class classification system. The six-class tumor subtyping (96.36%), stone detection (91.96%) and MRI-based CKD evaluation (93.85%) presented here present more complex diagnostic tasks, which use different medical imaging methods, than standard reference points. The three settings produce results that are less accurate than coarse CT classification, but they help researchers understand how well models perform under various conditions. These more challenging settings yielded lower accuracy than conventional CT classification tasks, but provided a more informative assessment of model performance under heterogeneous conditions. As shown in Table 7, many contemporary architectures achieve similarly high performance on established CT benchmarks. In this context, the main value of HMPA-CNN lies less in marginal gains on saturated datasets than in its more consistent performance across diverse renal imaging tasks, datasets, and modalities within a unified framework.

## 5. Conclusions

This study evaluated a dual-path attention-based convolutional architecture for kidney disease classification across five independent datasets spanning tumor characterization, stone detection, and chronic kidney disease assessment using both CT and MRI. High accuracy was observed in three- and four-class tumor classification settings, while performance decreased under fine-grained subtype differentiation, small-object detection, and cross-modality evaluation. These findings reflect increased task complexity and highlight performance variability across different diagnostic settings.

The architecture maintained stable performance across geographically and institutionally distinct CT cohorts, suggesting resilience to variations in acquisition protocols and scanner types. The explicit separation of spatial (3 × 3) and textural (5 × 5) feature extraction, followed by attention-based recalibration and gated fusion, provides a structured alternative to monolithic convolutional or transformer-based designs. Consistently high specificity in tumor classification tasks indicates potential utility in reducing false-positive interpretations in screening or triage contexts.

At the same time, the reduction in performance for kidney stone detection and six-class tumor subtyping highlights intrinsic diagnostic challenges and architectural limitations. Cross-modality evaluation on MRI-based CKD classification demonstrated preserved discriminative capacity, although lower than that of CT-based mass classification.

Collectively, these findings support the potential of multi-path attention mechanisms in renal imaging, but remain constrained by the retrospective design, reliance on curated datasets, and performance variability in more challenging tasks. Prospective multicenter validation is therefore required before clinical translation.

### 5.1. Limitations

All analyses were performed retrospectively on curated, expert-annotated datasets, without prospective validation or systematic correlation with histopathological findings. The impact of the model on clinical decision-making was not assessed. Prospective validation, reader studies, and testing under routine clinical workflow conditions remain necessary before translation into practice. Future reader studies should include larger image sets, multiple clinical centers, and inter-rater agreement analysis to more rigorously compare model performance with expert interpretation. In addition, the framework relies on 2D slice-based analysis and does not incorporate full volumetric context. Although multiple datasets were included, several experiments were conducted on public benchmarks that may not fully reflect the heterogeneity of routine clinical practice. Although the model showed fast image-level inference in the present setting, computational performance may vary across hardware platforms and deployment environments.

### 5.2. Future Work

Future research should prioritize multicenter studies conducted in real clinical environments. Combining volumetric modeling with multi-sequence integration may improve understanding of complex lesions. The model needs to assess which specific architectural changes affect the ability to detect small objects. The system needs future research to determine its ability to make accurate measurements, its operational stability when acquiring data from different situations and its potential to work with human–AI collaborative diagnostic systems.

## Figures and Tables

**Table 1:** Performance metrics for the KidneyNeXt dataset.

Class	Precision (%)	Recall (%)	Specificity (%)	F1-Score (%)	Accuracy (%)
Benign	99.74	99.74	99.78	99.74	99.76
Control	100.00	100.00	100.00	100.00	100.00
Malignant	99.56	99.56	99.84	99.56	99.76
Macro Avg	99.77	99.77	99.87	99.77	99.84
Weighted Avg	99.76	99.76	99.86	99.76	99.83
Overall Accuracy	-	-	-	-	99.76

**Table 2:** Performance metrics for four-class CT classification.

Class	Precision (%)	Recall (%)	Specificity (%)	F1-Score (%)	Accuracy (%)
Control	100.00	100.00	100.00	100.00	100.00
Cyst	99.87	100.00	99.94	99.93	99.96
Stone	100.00	99.64	100.00	99.82	99.96
Tumor	100.00	100.00	100.00	100.00	100.00
Macro Avg	99.97	99.91	99.99	99.94	99.98
Weighted Avg	99.96	99.96	99.98	99.96	99.98
Overall Accuracy	-	-	-	-	99.96

**Table 3:** Performance metrics for four-class classification in the Kauh Jordan dataset.

Class	Precision (%)	Recall (%)	Specificity (%)	F1-Score (%)	Accuracy (%)
Benign	99.81	99.62	99.90	99.72	99.81
Control	100.00	100.00	100.00	100.00	100.00
Cyst	99.29	99.64	99.84	99.47	99.81
Malignant	99.68	99.68	99.92	99.68	99.87
Macro Avg	99.69	99.74	99.92	99.71	99.87
Weighted Avg	99.75	99.74	99.92	99.74	99.87
Overall Accuracy	-	-	-	-	99.74

**Table 4:** Performance metrics for six-class subtype classification.

Class	Precision (%)	Recall (%)	Specificity (%)	F1-Score (%)	Accuracy (%)
Adenoma	95.90	95.41	98.61	95.65	97.79
Angiomyolipoma	91.30	100.00	99.15	95.45	99.22
Control	95.86	98.21	98.26	97.02	98.25
Cyst	99.21	93.98	99.84	96.53	98.83
RCC	96.55	90.91	99.64	93.65	98.77
Secondary	98.72	100.00	99.86	99.35	99.87
Macro Avg	96.26	96.42	99.23	96.28	98.79
Weighted Avg	96.43	96.36	98.99	96.36	98.53
Overall Accuracy	-	-	-	-	96.36

**Table 5:** Performance metrics for kidney stone detection.

Class	Precision (%)	Recall (%)	Specificity (%)	F1-Score (%)	Accuracy (%)
Non-Stone	90.62	94.68	88.89	92.60	91.96
Stone	93.65	88.89	94.68	91.21	91.96
Macro Avg	92.13	91.78	91.78	91.90	91.96
Weighted Avg	92.04	91.96	91.60	91.95	91.96
Overall Accuracy	-	-	-	-	91.96

**Table 6:** Performance metrics for MRI-based CKD classification.

Class	Precision (%)	Recall (%)	Specificity (%)	F1-Score (%)	Accuracy (%)
CKD	90.14	98.46	89.23	94.12	93.85
Control	98.31	89.23	98.46	93.55	93.85
Macro Avg	94.22	93.85	93.85	93.83	93.85
Weighted Avg	94.22	93.85	93.85	93.83	93.85
Overall Accuracy	-	-	-	-	93.85

**Table 7:** Comparative performance of recent deep learning approaches for kidney disease.

Study	Methodology	Number of Samples	Results (%)
Alzu’bi et al. (2022) [24]	2D CNN, ResNet50, VGG16	4800 CT images (210 patients)	Accuracy: 97.00 (CNN-6); 96.00 (ResNet50); 60.00 (VGG16)
Khan (2025) [30]	ConvLSTM + Inception + feature fusion	2886; Dataset2: 12,446	Accuracy: 99.30; Precision: 98.00; Recall: 100.00; F1-score: 99.00/Cross-dataset accuracy: 91.31
Rehman et al. (2025) [31]	Swin ViT + DeepLabV3+ + transfer learning	Dataset2: 12,446 (5077 Normal, 3709 Cyst, 2283 Tumor, 1377 Stone)	Accuracy: 99.20; F1-score: 99.50; Precision: 99.10; Specificity: 99.30, AUC:0.997
Prabhu et al. (2025) [32]	ProsGradNet	Prostate: 11,684 train/2854 test/2939 val; KMC: 3432 train/506 test/503 val	Accuracy: 92.88 (Prostate); 92.68 (KMC); F1-score: 92.63–92.92
Yan et al. (2025) [33]	LRCTNet	3090 from 318 patients	Accuracy: 95.79; Precision: 93.91; Recall: 93.48; F1-score: 93.70; MCC: 94.38, AUC:98.62
Ayogu et al. (2025) [34]	Ensemble (InceptionV3, CCT, SwinT, VGG16, ResNet50)	Dataset2: 12,446 (5077 Normal, 3709 Cyst, 2283 Tumor, 1377 Stone)	Accuracy: 99.67; Precision: 99.10; Recall: 100.00
Shanmathi et al. (2025) [35]	Deep Neural Network (DNN)	Dataset2: 12,446 (5077 Normal, 3709 Cyst, 2283 Tumor, 1377 Stone)	Accuracy: 96.31; Precision: 94.10; Recall: 96.31
Kashyap et al. (2025) [36]	Custom CNN, ViT, VGG19, ResNet50	Dataset2: 12,446 (Train: 10,955; Test: 1249; Val: 1242)	Accuracy: 99.60 (ViT-TL); 98.48 (ResNet50); 98.16 (CNN); 94.64 (VGG19)
Kulandaivelu et al. (2025) [37]	AMC-AM + MSD-CMPA	-	Accuracy: 95.44; Precision: 95.13; Recall: 95.44; F1-score: 95.29; MCC: 90.87
Hossain et al. (2025) [38]	EfficientNet-B7 + ROI + pixel reduction	12,446 (5077 Normal, 3709 Cyst, 2283 Tumor, 1377 Stone)	Accuracy: 99.75; Precision: 98.45; Recall: 99.02; F1-score: 98.78; AUC: 95.78
Kulkarni et al. (2025) [39]	Hybrid ResNet-50 + Vision Transformer	9410 (5915 Normal, 3495 Stone)	Accuracy: 99.50; Comparative accuracy: 99.36 (XResNet); 98.69 (MobileNet); 98.13 (SwinT)
Pimpalkar et al. (2025) [11]	Fine-tuned CNNs (VGG16, ResNet50, AlexNet, InceptionV3)	Dataset2: 12,446 (5077 Normal, 3709 Cyst, 2283 Tumor, 1377 Stone)	Accuracy: 99.96 (InceptionV3); 100.00 (VGG16, AlexNet); 99.85 (ResNet50)
Zain et al. (2025) [40]	CGPCAP (Canny + GLCM + PCA) + CNN	200 (Train: 160, Test: 40)	Accuracy: 97.50; Precision: 93.75; Recall: 93.75; F1-score: 93.75; Specificity: 98.43
Chaki & Uçar (2024) [41]	DarkNet19, InceptionV3, ResNet101 + Ensemble + KNN + Bayesian CV	Dataset2: 12,446 (5077 Normal, 3709 Cyst, 2283 Tumor, 1377 Stone)	Accuracy: 99.80% (clean), 96.70% (noisy)
Maçin (2025) [22]	KidneyNeXt	Dataset1: 4199 (Train: 1535/906/918; Test: 384/227/229); Dataset2: 12,446 (Train: 11,948; Test: 1332/1828/502/818); Dataset3: 7770 (Train: 7216; Test: 532/266/308/448)	Dataset1—Accuracy 99.76%, Precision: 99.71%, Recall: 99.71%, F1-score: 99.71%. Dataset2—Accuracy 99.96%, Precision: 99.95%, Recall: 99.93%, F1-score: 99.94%. Dataset3—Accuracy 99.74%, Precision: 99.72%, Recall: 99.73%, F1-score: 99.72%
Londoño et al. (2025) [42]	Two deep learning approaches: (1) Custom CNN (3 Conv layers + MaxPooling + Dense 512 + Dropout 0.4; Adam optimizer, LR = 5 × 10^−4^), (2) Transfer Learning using MobileNetV2 (pretrained on ImageNet; last 30 layers trainable; AdamW optimizer, LR = 1 × 10^−5^); 70/30 train-validation split; grayscale preprocessing, edge masking, gamma correction	3364 original axial CT images	CNN Model: Accuracy: 94.8%, Precision: 96.5%, Recall: 92.2%, F1-score: 94.3%, AUC: 99.0% MobileNetV2: Accuracy: 94.4%, Precision: 94.1%, Recall: 93.9%, F1-score: 94.0%, AUC: 98.6%
Aljohani et al. (2025) [43]	Hierarchical AI framework using RAD-DINO-MAIRA-2 encoder (ViT-based feature extraction) + 32 ML classifiers ducibility	8400 CT images (120 patients; 60 tumor, 60 normal; 38 benign, 22 malignant; 70 images per case)	Overall (Mean over 25 trials): Accuracy: 94.72%, Precision: 93.29%, Recall: 94.02%, F1-score: 94.34%
Damiano et al. (2025) [44]	MRMR feature selection, Logistic Regression with leave-one-patient-out cross-validation	Dataset 5: 60 subjects (30 CKD, 30 HC)	AUC: GT: 99.84%, DP: 97.60%, MCD: 96.16%, TTA: 99.84%, MCDPLUS: 99.36%, TTAPLUS: 96.32%
Afnaan et al. (2024) [45]	Feature extraction using GLCM (20 features), DCT (1024 features), and DWT (256 features); classifiers: Logistic Regression (LR), SVM, Random Forest (RF), kNN, Decision Tree (DT), Naïve Bayes (NB); Soft Voting Ensemble	Dataset 5: 1010 T2-weighted MRI images (522 Healthy, 488 CKD); 80/20 train-test split	GLCM: LR Accuracy: 92%, Precision: 92%, Recall: 92%, F1: 92% DCT: SVM & RF Accuracy: 91.5%, Precision: 92%, Recall: 92%, F1: 92% DWT: RF Accuracy: 86.6% (best for DWT set) Soft Voting: GLCM: 90%, DCT: 89%, DWT: 84%
Tawfik et al. (2026) [46]	Pyramid Channel and Spatial Attention (PCSANet) (ResNet-based architecture with Pyramid Channel & Spatial Attention module, evaluated using 10-fold and 5-fold cross-validation)	12,446 CT images (5077 Normal, 3709 Cyst, 2283 Tumor, 1377 Stone)	Standard PCSA Integration: Accuracy: 99.92%, Precision: 99.9%, Recall: 99.92%, F1-score: 99.9% Pre-PCSA Integration: Accuracy: 96.24%, Precision: 95.88%, Recall: 96.24%, F1-score: 96.02% Post-PCSA Integration: ~97% Accuracy, ~97% Precision, ~97% Recall, ~97% F1-score
Gujarathi-Mehta et al. (2026) [47]	Spinal Convolutional Neural Network (Spinal CNN) (Hybrid model combining CNN + SpinalNet; feature extraction: shape, statistical, and textural features; evaluated using hold-out (90% training) and 9-fold cross-validation)	12,446 CT images (5077 Normal, 3709 Cyst, 2283 Tumor, 1377 Stone)	CT Kidney Dataset (90% training): Accuracy: 90.3%, Sensitivity: 91.8%, Specificity: 89.9% CT Kidney Dataset (9-fold CV): Accuracy: 87.4%, Sensitivity: 88.5%, Specificity: 89.5%
**Our Study**	HMPA-CNN Hybrid Multi-Path Attention CNN (dual-path spatial 3 × 3 + texture 5 × 5 branches), Attention-Inspired Feature Recalibration Modules (AFRM), gated feature fusion, Multi-Branch Feature Aggregation Module (MFAM), adaptive feature recalibration; Adam optimizer (LR = 0.0001, decay schedule)	Dataset 1 (KidneyNeXt): 4199 CT images (3-class) Dataset 2: 12,446 CT images (4-class) Dataset 3 (KAUH 4-class): 7840 CT images Dataset 3 (KAUH 6-class): 7700 CT images Dataset 4 (Stone): 3364 CT images (binary) Dataset 5 (CKD MRI): 1299 (MRI)	Dataset 1 (3-class): Acc: 99.76%, Prec: 99.77%, Rec: 99.77%, F1: 99.77%, AUC:0.99 Dataset 2 (4-class): Acc: 99.96%, Prec: 99.97%, Rec: 99.91%, F1: 99.94%, AUC 0.99, Dataset 3 (4-class): Acc: 99.74%, Prec: 99.69%, Rec: 99.74%, F1: 99.71%, AUC 0.99, Dataset 3 (6-class): Acc: 96.36%, Prec: 96.26%, Rec: 96.42%, F1: 96.28% Dataset 4 (Stone): Acc: 91.96%, Prec: 92.13%, Rec: 91.78%, F1: 91.90%, AUC 0.92. Dataset 5 (CKD MRI): Acc: 93.85%, Prec: 94.22%, Rec: 93.85%, F1: 93.83%, AUC: 0.94

## Data Availability

The datasets analyzed in this study are publicly available from the original sources cited in the References section. No new data were generated.
